# Genome-Wide Identification and Expression of *FAR1* Gene Family Provide Insight Into Pod Development in Peanut (*Arachis hypogaea*)

**DOI:** 10.3389/fpls.2022.893278

**Published:** 2022-05-03

**Authors:** Qing Lu, Hao Liu, Yanbin Hong, Xuanqiang Liang, Shaoxiong Li, Haiyan Liu, Haifen Li, Runfeng Wang, Quanqing Deng, Huifang Jiang, Rajeev K. Varshney, Manish K. Pandey, Xiaoping Chen

**Affiliations:** ^1^Guangdong Provincial Key Laboratory of Crop Genetic Improvement, Crops Research Institute, Guangdong Academy of Agricultural Sciences, South China Peanut Sub-Center of National Center of Oilseed Crops Improvement, Guangzhou, China; ^2^Key Laboratory of Biology and Genetic Improvement of Oil Crops, Ministry of Agriculture, Oil Crops Research Institute of the Chinese Academy of Agricultural Sciences, Wuhan, China; ^3^Center of Excellence in Genomics and Systems Biology, International Crops Research Institute for the Semi-Arid Tropics, Hyderabad, India; ^4^State Agricultural Biotechnology Centre, Centre for Crop and Food Innovation, Food Futures Institute, Murdoch University, Murdoch, WA, Australia

**Keywords:** peanut (*Arachis hypogaea*), genome-wide, far-red-impaired response 1 (FAR1), pod development, expression pattern

## Abstract

The *far-red-impaired response 1* (*FAR1*) transcription family were initially identified as important factors for phytochrome A (phyA)-mediated far-red light signaling in *Arabidopsis*; they play crucial roles in controlling the growth and development of plants. The reported reference genome sequences of *Arachis*, including *A. duranensis*, *A. ipaensis*, *A. monticola*, and *A. hypogaea*, and its related species *Glycine max* provide an opportunity to systematically perform a genome-wide identification of *FAR1* homologous genes and investigate expression patterns of these members in peanut species. Here, a total of 650 *FAR1* genes were identified from four *Aarchis* and its closely related species *G. max*. Of the studied species, *A. hypogaea* contained the most (246) *AhFAR1* genes, which can be classified into three subgroups based on phylogenic relationships. The synonymous (*Ks*) and non-synonymous (*Ka*) substitution rates, phylogenetic relationship and synteny analysis of the *FAR1* family provided deep insight into polyploidization, evolution and domestication of peanut *AhFAR1* genes. The transcriptome data showed that the *AhFAR1* genes exhibited distinct tissue- and stage-specific expression patterns in peanut. Three candidate genes including *Ahy_A10g049543*, *Ahy_A06g026579*, and *Ahy_A10g048401*, specifically expressed in peg and pod, might participate in pod development in the peanut. The quantitative real-time PCR (qRT-PCR) analyses confirmed that the three selected genes were highly and specifically expressed in the peg and pod. This study systematically analyzed gene structure, evolutionary characteristics and expression patterns of *FAR1* gene family, which will provide a foundation for the study of genetic and biological function in the future.

## Introduction

Plants, as sessile organisms, utilize sophisticated sensory systems to adapt to environmental changes. As one of the most important environmental factors, light participates in multiple biological processes, such as plant growth, photomorphogenesis, stomata movement, chloroplast development, circadian rhythms, and flowering (Quail, 2002; Wang and Deng, 2003; Huang et al., 2012; Huai et al., 2020; Krzeszowiec et al., 2020). In order to adapt to different light environment changes, such as direction, duration, quantity, and wavelength of light, plants have obtained a series of sophisticated photoreceptors, including phytochromes, cryptochromes, and phototropins receptors (Casal, 2000). The phytochromes mainly sense red and far-red wavelengths whereas the cryptochromes and phototropins detect the blue/ultraviolet-A region of the spectrum. Of them, phytochromes are the most extensively studied. In *Arabidopsis*, five distinct genes (*PHYA*-*PHYE*) encoding specific phytochromes were identified (Briggs and Olney, 2001). Of the five phytochromes, PHYA is primarily responsible for eliciting various far-red light-mediated responses, including regulation of hypocotyl elongation and controlling flowering and expansion of cotyledons (Whitelam et al., 1993; Briggs and Olney, 2001). The energetic activity of PHYA is transported from the cytosol to the nucleus to mediate various far-red light induced responses through interactions with far-red elongated hypocotyls 1 (FHY1) or FHY1-like (FHL) proteins, whose products are crucial for PHYA nuclear accumulation. Subsequently, the nuclear accumulation of PHYA promotes downstream transcription and activates the subsequent responses (Wang and Deng, 2002; Lin et al., 2007). Molecular studies have confirmed that *far*-red elongated *hypocotyl 3* (*FHY3*) and its homologous gene *far*-red-impaired response 1 (*FAR1*), which encode two proteins related to *Mutator*-like transposases, directly activate the transcription of *FHY1/FHL* to modulate PHYA signaling under far-red light conditions by binding the promoters of *FHY1* and *FHL*. Consequently, *FHY3* and *FAR1* also play multifaceted roles in PHYA signaling (Wang and Wang, 2015).

In *Arabidopsis*, *FHY3*, *FAR1*, and 12 *FAR1*-related sequences (*FRS1* to *FRS12*) have been identified. These genes or sequences revealed high homology and highly conserved protein secondary structures (Lin and Wang, 2004). A phylogenetic analysis of proteins indicated that the *FHY3* and *FAR1* family can be classified into six subgroups. Most of the families of proteins have a DNA-binding domain in their N-terminal regions. As exceptions, FRS7 and FRS12 have two DNA-binding domains, and FRS9 has no DNA-binding domain in the N-terminal region (Ma and Li, 2018). In addition, FRSs and FRS-like proteins have also been identified in other plants, suggesting that these proteins are conserved throughout the evolution of the plant kingdom (Takano et al., 2001; Du et al., 2021). Chromatin immunoprecipitation sequencing analyses have indicated that FHY3 specifically binds to the promoter regions of 1559 and 1009 direct target genes in dark and far-red light conditions, respectively, through the FHY3/FAR1 binding motif (CACGCGC) in *Arabidopsis* (Ouyang et al., 2011). Although FRS9 contains no DNA-binding domain, it might be involved in light signal transduction by interacting with other target genes. Gene expression regulation studies indicated that *FHY3/FAR1* play a critical role in light signal transduction, and regulates plant growth, development, immunity, and defense (Wang et al., 2016; Liu et al., 2019b; Xie et al., 2020).

Peanut or groundnut (*Arachis hypogaea*) is one of the most important oil and food leguminous crops, which was expanded to Europe, Africa, Asia, and the Pacific Islands and has an annual production of ∼53.6 million tons in 2020 (FAOSTAT., 2020). Peanut is an allotetraploid (AABB, 2n = 4x = 40) derived from hybridization between two diploids progenitors, *A. duranensis* (AA) and *A. ipaensis* (BB), which all have been sequenced previously (Seijo et al., 2007; Robledo et al., 2009; Bertioli et al., 2016; Chen et al., 2016a; Lu et al., 2018). Genomic *in situ* hybridization indicated that the allotetraploid wild species *A. monticola* (AABB, 2n = 4x = 40) might be the immediate wild ancestor of *A. hypogaea* (Seijo et al., 2007). Peanut is distinguished from other crops that flower by aerial methods and form subterranean fruits. After flowering, self-pollination and fertilization, the gynophore (commonly called peg) carrying the embryo elongates into to the ground to penetrate the soil to develop into a pod, under dark conditions. However, when the gynophore fails to penetrate into the soil, the embryo is affected by light, resulting in abortion of the embryo formation. Therefore, light plays a crucial determining role in regulating embryo development and promoting pod enlargement in peanut. The *FAR1* gene has been observed to play key roles in light signal transduction and regulation of plant development. In peanut species, functional analysis of *AhJ11-FAR1-5* indicated that this gene enhanced tolerance to drought stress (Yan et al., 2020). However, there are several reports of the *FAR1* gene family being involved in light responses in peanut. Interestingly, we identified that the *FAR1* transcription factors expanded in cultivated peanut and its two wild diploid progenitors, *A. duranensis* and *A. ipaensis* (Chen et al., 2016a; Lu et al., 2018). We hypothesized that the *Arachis*-specific expansion of the *FAR1* family may be related to geocarpy and pod development, considering the pivotal role of the *FAR1* gene family in modulating phyA-signaling transduction in plants (Casal, 2000; Wang and Deng, 2003; Huang et al., 2012). Herein, a genome wide identification of *FAR1* gene family in *Arachis*, including *A. duranensis*, *A. ipaensis*, *A. monticola*, and *A. hypogaea*, and its closely related species *G. max* was performed to interrogate gene structure, evolutionary relationships, conserved motifs, and expression patterns. This study will provide a theoretical reference for further genetic and functional studies.

## Materials and Methods

### Identification of the *FAR1* Family Members in *Arachis* and *Glycine max*

The HMMER3 (Mistry et al., 2013)^1^ was used to search *FAR*1 gene family domains based on the hidden Markov model (HMM) file (PF03101), which was downloaded from the Pfam (Finn et al., 2014) database^2^ by the *FAR1* DNA binding domain identity PF03101. Four *Arachis* genomes, including *A. duranensis*, *A. ipaensis* (Bertioli et al., 2016), *A. monticola* (Yin et al., 2018), and *A. hypogaea* (Chen et al., 2019), were downloaded from the relevant database exhibited in each previous report. The *G. max* genome version 2.1 was obtained from the NCBI database (assembly accession: GCF_000004515.5). The key parameters were set as default and the cutoff value was set as 1e-15. The identified *FAR1* members were confirmed using NCBI-Conserved Domain Database (CDD),^3^ SMART,^4^ and Pfam (Finn et al., 2014) (see text footnote 2) databases. All incorrect, repetitive, and non-*FAR1* family members were removed.

### Sequence Alignment and Phylogenetic Tree

The protein sequences of *FAR1* family members were used to create multiple alignments using ClustalW with default parameter sets. The phylogenetic tree was constructed using MEGA version 7.0 under the neighbor-joining (NJ) method with 1000 bootstrap replicates (Kumar et al., 2016).

### Gene Distribution and Duplication

According to physical position, all the *FAR1* genes were mapped to each reference genome using TBtools version 1.068 (Chen et al., 2020). Gene duplication events were analyzed using MCScanX with default parameters (Wang et al., 2012). The syntonic map was constructed using Python jcvi utility libraries.^5^ Non-synonymous (*Ka*) and synonymous (*Ks*) substitution of duplicated *FAR1* genes were calculated by using KaKs_Calculator 2.0 (Wang et al., 2010) and plotted using R ggplot2 (Wickham, 2016).

### Gene Structure, Conserved Domain, and *cis*-Acting Elements

The *FAR1* gene structures were analyzed based on reference genome annotation information using our in-house Perl scripts. The conserved domain motifs of FAR1 members were identified by MEME version 4.12.0 (Bailey et al., 2015) with the number of motifs set to 10 and minimum and the maximum length of motifs set to six and 100, respectively (Bailey et al., 2009). The results of the gene structures, conserved domains and phylogenetic tree were plotted by using TBtools version 1.068 (Chen et al., 2020). The *in silico* elements of the *AhFAR1* promoter in 1500 bp regions were searched using the PlantCARE database (Lescot et al., 2002).^6^ The original results were filted and “light responsive” elements were retained. The features of *in silico* elements were visualized by GSDS 2.0 (Hu et al., 2015).^7^

### Transcriptome and Quantitative Real-Time PCR Analysis

The transcriptomes of five different typical tissues, including flower, peg, leaf, root, and stem, were obtained from our previous study to analyses the expression levels of the members of the *AhFAR1* gene family (Chen et al., 2019). Moreover, the transcriptomes of 11 development stages of shell and seed were downloaded from our previous report to evaluate the *AhFAR1* gene expression patterns in underground tissues (Chen et al., 2016b). The expression levels of the *AhFAR1* were evaluated using the fragment per kilobase million (FPKM) method. The expression levels were visualized using FPKM standardization data in R pheatmap packages.^8^

Six different typical components of peanut, including root, stem, leaf, peg, and pod, were used to verify *AhFAR1* gene expression levels. In addition, five different developmental stages of ovule-carrying peg (peg length = 1–5 cm with 1 cm as step) and pods (including aerial, not swelling subterranean, early swelling subterranean, swelling subterranean, and mature pods) were used to confirm the expression levels of candidate *AhFAR1* genes. Total RNA was extracted from each sample using a Plant RNeasy Mini Kit (TIANGEN, Beijing, China). The quality of the RNA extracted from each sample was checked using a Nano Drop (Thermo Scientific, United States). The qRT-PCR was carried out as described by Liu et al. (2019a). DNA-free RNA was used to synthesize the first strand of cDNA. The *yellow leaf specific 8* (*yls8*) gene (Forward Primer: 5′-AACTGCTTAGCTGCTATTACCC-3′, Reverse Primer: 5′-TCGCCAAATAACACGTTGCATT-3′) was used as an internal control. Each measurement was carried out in three experimental replications, and each reaction was performed in biological triplicate replications. The relative expression levels of each target gene was analyzed using 2^–△^^△^ *^CT^* method.

## Results

### Identification of *FAR1* Genes in *Aarchis* and *Glycine max*

**FIGURE 1:**
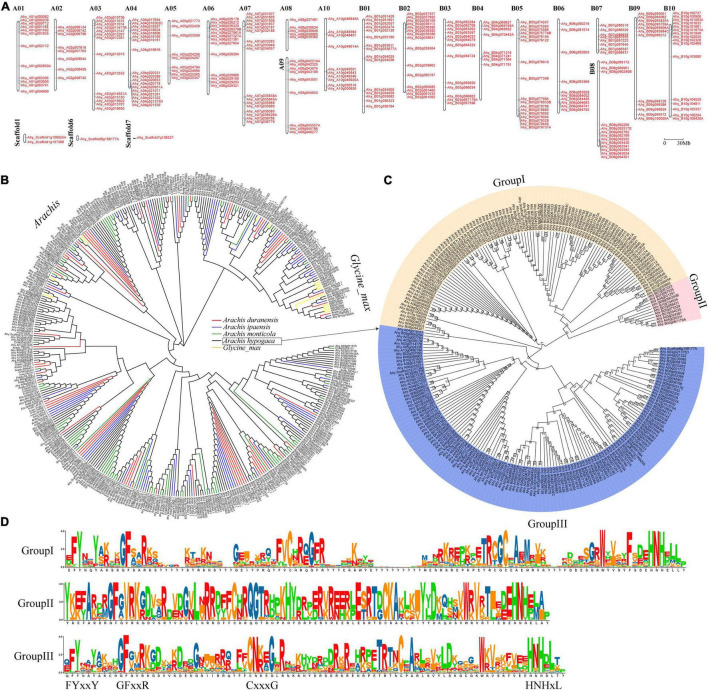
Genome-wide distribution and phylogenetic tree of AhFAR1 domains. **(A)** Distribution of the 246 *AhFAR1* genes on peanut 20 chromosomes and 3 scaffolds. **(B)** Phylogenetic tree of FAR1 domains of different *Arachis* and *G. max*. **(C)** Phylogenetic tree of 246 AhFAR1 domains of *A. hypogaea*. The numbers at the nodes represent bootstrap percentage values computed by 1,000 replications. **(D)** Motif analysis of three subgroups of 246 AhFAR1.

A total of 650 *FAR1* genes were identified from four *Aarchis* and its closely related species *G. max* (Supplementary Table 1). Of them, the most (246) and least (36) *FAR1* genes were retrieved from *A. hypogaea* and *G. max*, respectively. The total number of *FAR1* genes identified from each genome exhibited great consistency with the evolutionary genetic relationships (Supplementary Figure 1). This result suggested that the *FAR1* gene family was expanding rapidly in the *Arachis* species, which was consistent with our previous findings (Chen et al., 2016a; Lu et al., 2018; Yan et al., 2020). All of the identified *FAR1* genes were mapped on the chromosomes of their respective genomes based on the physical positions of the genes (Figure 1A and Supplementary Figure 2).

The phylogenetic analysis revealed that the FAR1 domains could be roughly divided into *Arachis* and *G. max* groups. However, these domains identified in *Arachis* species could not be clearly clustered by different species attributes (Figure 1B). Moreover, the FAR1 identified in different species could be classified into different corresponding groups (Supplementary Figure 3). Deep phylogenetic analysis of the 246 AhFAR1 domains identified in *A. hypogaea* indicated that these domains could be classified into three main groups. Among them, 99, 11, and 136 members belong to group I, II, and III, respectively (Figure 1C). The protein sequence analysis indicated that the conserved motifs of the domains in each group were different, although they all contained the typical FAR1 conserved motifs, for example the N-terminus contained FYxxY, GFxxR, and CxxxG and the C-terminus had HNHxL (Figure 1D).

### Gene Structure, Conserved Motif, and Promoter Analyses

**FIGURE 2:**
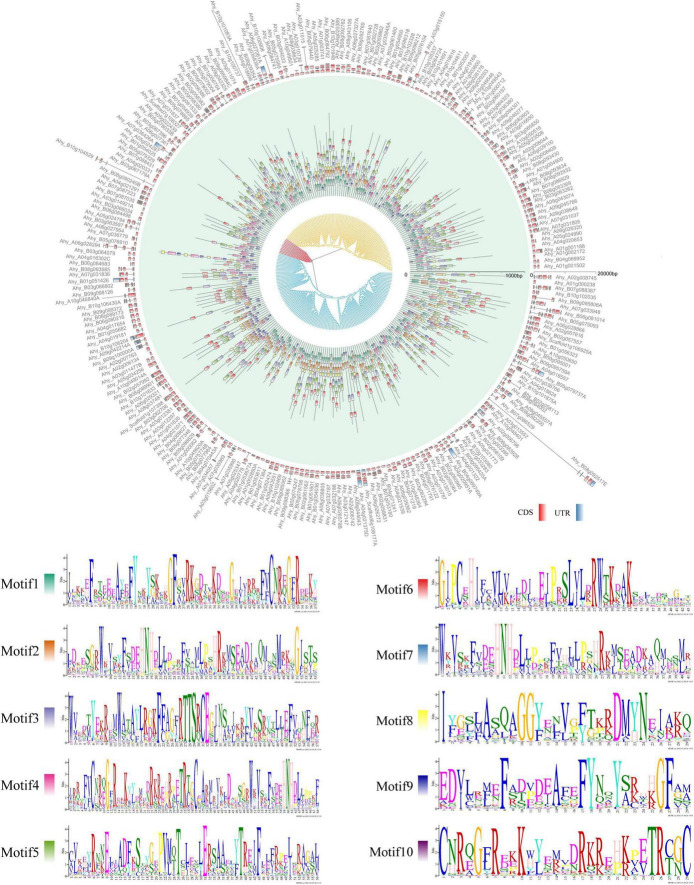
Comparative gene structure, phylogenetic tree, and motif analysis of *AhFAR1* genes. Inner, middle, and outer circle represent phylogenetic tree, motif, and gene structure, respectively.

The gene structure (exon-intron-UTR), conserved motif organization and phylogenetic tree of all the *FAR* genes identified in each species were analyzed to gain insight into the difference of the *FAR* family in different plant species (Figure 2 and Supplementary Figure 4). The results showed that members belonging to the same group had almost similar motifs and gene structures. For example, in *A. duranensis*, the members of the red group typically contained motif 3 and motif 8. The special motif 10 gene was observed in blue group (Supplementary Figure 4). In *A. hypogaea*, the distribution of exon-intron-UTR structure and the motif phase corresponded with the clusters of *AhFAR1* genes (Figures 1C, 2), especially for group I (yellow) and III (blue), most of which contains motif 1 and motif 2. However, for group II (red) the genes did not contain these two motifs, but contained motif 3 and motif 4 instead (Figure 2). Overall, the conserved motif organizations, similarity of gene structures and phylogenetic tree results could be confidently verified, confirming the reliability of the classifications and evolutions of the *FAR1* gene family in different leguminous plant species.

In plants, *cis*-acting elements of promoter regions often affect the biological functions of genes. Here, in order to identify the *cis*-acting elements of the 246 *AhFAR1* genes obtained from *A. hypogaea* genome, a 1.5 Kb upstream genomic region of each gene was explored in the PlantCARE database (Lescot et al., 2002). A total of 2,147 *cis*-acting elements related to “light responsiveness” were identified (Supplementary Table 2 and Supplementary Figure 5). The average number of *cis*-acting elements identified by each gene was 8.7 (from 2 to 18). In *Ahy_A03g015787* and *Ahy_B02g058113*, 18 *cis*-acting elements were identified (Supplementary Table 3). Moreover, a total of 31 types of *cis*-acting elements were obtained. Among them, the Box-4 element was the most widely distributed (601), and the ACA-motif, Pc-CMA2a, CAG-motif and P-box were each only identified once (Supplementary Table 4). Functional annotation indicated that all of the obtained *cis*-acting elements, such as Box-4, G-box and GT1-motif, were identified to be involved in the photopic response system (Supplementary Table 2). All of these *cis*-acting elements were functional annotated as light responsiveness through different regulation patterns, such as conserved DNA module, MYB binding site, gapA-CMA1, and gibberellin pathway (Supplementary Table 2). The results regarding of the *cis*-acting elements suggested that the *AhFAR1* gene family could play critical roles in peanut growth and development.

### Evolutionary Selection Pressure and Synteny Analysis

**FIGURE 3:**
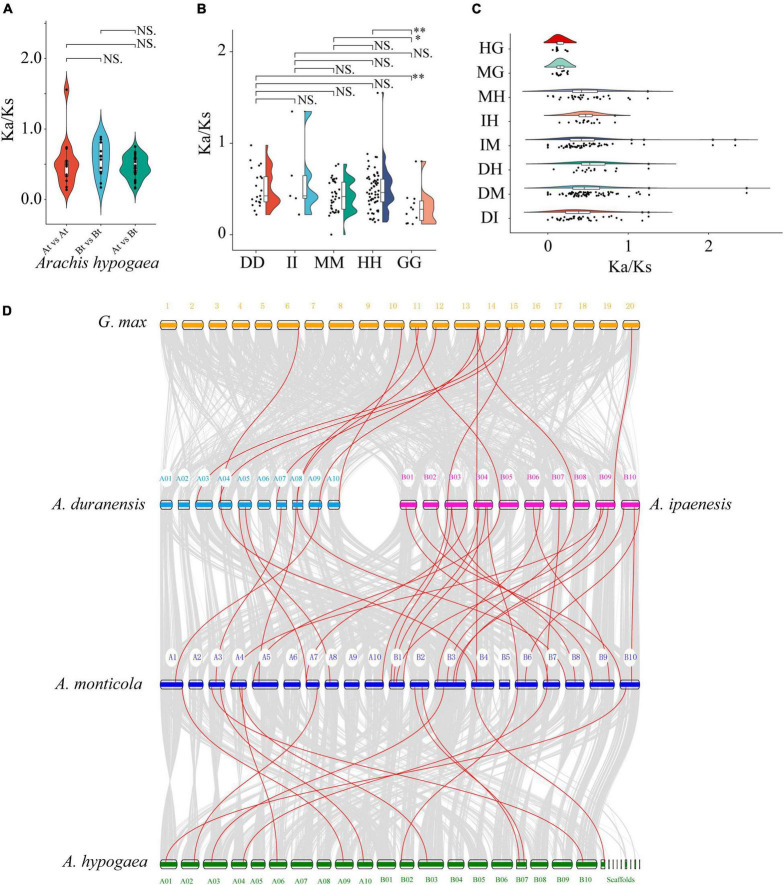
The *Ka/Ks* and synteny analyzes of *AhFAR1* genes among different plant species. **(A)** The comparison of *Ka/Ks* paralogous gene pairs within and between At and Bt. **(B)** The comparison of *Ka/Ks* paralogous gene pairs among *Arachis* and *G. max*. DD, *A. duranensis*; II, *A. ipaensis*; MM, *A. monticola*; HH, *A. hypogaea*; GG, *G. max*. **(C)** The comparison of *Ka/Ks* orthologous gene pairs among *Arachis* and *G. max*. HG, *A. hypogaea* vs. *G. max*; MG, *A. monticola* vs. *G. max*; MH, *A. monticola* vs. *A. hypogaea*; IH, *A. ipaensis* vs. *A. hypogaea*; IM, *A. ipaensis* vs. *A. monticola*; DH, *A. duranensis* vs. *A. hypogaea*; DM, *A. duranensis* vs. *A. monticola*; DI, *A. duranensis* vs. *A. ipaensis*. **(D)** Synteny plot of *AhFAR1* genes. Gray lines represent the collinear blocks between two plants, and red lines represent the syntenic *AhFAR1* gene pairs. NS. represents no significant at 0.05 significance level. * and ^**^ indicate 0.05 and 0.01 significance level, respectively.

The synonymous (*Ks*) and non-synonymous (*Ka*) substitution rates of paralogous and orthologous gene pairs were calculated to study selection pressures of *FAR1* genes in *Aarchis* and its related species *G. max* in the process of biological evolution. Each of the 13 paralogous gene pairs were identified in subgenome A (At) and subgenome B (Bt), respectively. Moreover, a total of 33 paralogous gene pairs were observed between At and Bt (Supplementary Table 5). There was no significant difference in *Ka/Ks* value either within or between the two subgenomes (Figure 3A), suggesting that the paralogous *AhFAR1* genes had undergone a relaxed selection pressure. Furthermore, a total of 11, 6, 21, 40, and 62 *FAR1* paralogous gene pairs were identified in *G. max*, *A. ipaensi*, *A. duranensiss*, *A. monticola*, and *A. hypogaea*, respectively (Supplementary Table 6). A large number of paralogous gene pairs were identified in the allotetraploid *A. hypogaea*, which might be caused by polyploidization or whole genome duplication events during evolution. The average *Ka/Ks* of paralogous *FAR1* in *G. max* (0.30) was significantly smaller than that calculated for *A. duranensiss* (0.51), *A. monticola* (0.42), and *A. hypogaea* (0.49). However, there was no significant difference in the average *Ka/Ks* value among the four *Aarchis* species (Figure 3B). These results indicated that the *FAR1* gene family experienced strong evolutionary selection pressure when evolution occurred from Leguminosae, while these genes were under relaxed selection in the subsequent evolution of *Arachis* species. The average *Ka/Ks* of orthologs in both *G. max* vs. *A. hypogaea* and *G. max* vs. *A. monticola* were all smaller than that of the *Arachis* combination gene pairs, suggesting that the biological functions of the *FAR1* gene family underwent a relaxed diversification after the divergence in *Arachis* species (Figure 3C and Supplementary Table 7).

According to the collinearity analyzes among *G. max* and *Arachis* species, only 13 *FAR1* gene pairs showed a syntenic relationship in both *G. max* vs. *A. duranensis* (8) and vs. *A. ipaenesis* (5), which was significantly less than that identified among *Arachis* plants (50) (Figure 3D and Supplementary Table 8). This result suggested that the *FAR1* gene family was substantially expanded during polyloidization, evolution, and domestication. In addition, many more *FAR1* homologous gene pairs were identified among *A. monticola* vs. *A. duranensis* (8) and vs. *A. ipaenesis* (25) than that of among *A. hypogaea* vs. *A. duranensis* (2) and vs. *A. ipaenesis* (8) (Supplementary Table 8 and Supplementary Figure 6). This finding might support the conclusion that *A. hypogaea* was domesticated from an intermediate species, the wild tetraploid *A. monticola*, which was formed from its two diploid progenitors, *A. duranensis* and *A. ipaenesis* (Yin et al., 2019). Interestingly, some *FAR1* genes were associated with at least three or more syntenic gene pairs, such as *Araip.M4D5S* displaying collinearity with *GLYMA_13G341600*, *EVM0033853*, and *Aradu.8P3KF* in different plants (Figure 3D and Supplementary Table 8), indicating that these genes play key roles in the *FAR1* gene family and were highly conserved during evolution.

### Expression Analysis of *AhFAR1* Genes in *Arachis hypogaea*

**FIGURE 4:**
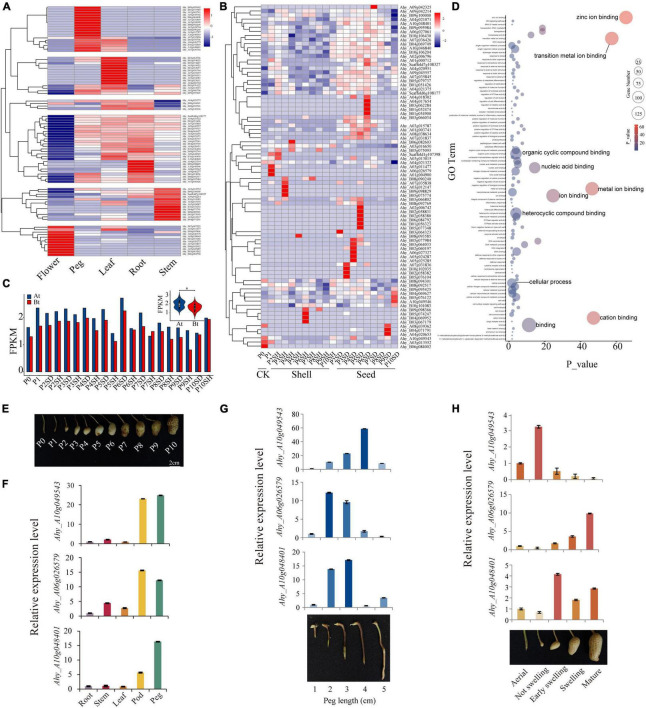
Expression patterns of the peanut *AhFAR1* gene family. **(A)** Clustering of expression profiles of *AhFAR1* genes in different tissues, such as flower, peg, leaf, root, and stem. **(B)** Clustering of expression profiles of *AhFAR1* genes in shell and seed at 11 development stages. **(C)** Comparison of the *AhFAR1* expression levels between At and Bt. **(D)** GO term enrichment analysis of the *AhFAR1* genes. **(E)** Eleven different developmental stages of peanut pods. **(F–H)** Relative expression level analyses of three candidate *AhFAR1* genes in representative tissues, different length of pegs, and development stages of pod. *represents *t*-test of *P* < 0.05.

In order to investigate the expression patterns of *AhFAR1* in different tissues (flower, peg, leaf, root, and stem) and in the different development stages of the pod (11 stages of shell and seed), the transcriptome data obtained from our previous study (Chen et al., 2016b,^2019^) was re-interrogated. The results revealed that a total of 157 *AhFAR1* genes were not expressed in any detected tissue, which was speculated to be because these genes had other special expression patterns and could not be detected in our previous study (Supplementary Table 9). However, as showed in Figure 4A, a total of 89 *AhFAR1* genes showed tissue-specific expression in peanut. For example, 12 genes in flower, 18 genes in pod, 15 genes in leaf, 31 genes in leaf and root, and 13 genes in stem showed the highest expression levels of the different tissues. In depth analysis of the expression patterns of the 246 *AhFAR1* genes in 11 developmental stages of shell and seed showed that 84 of them were expressed in at least one development stage (Supplementary Table 10), and some of the 84 genes exhibited stage-specific expression in shell or seed (Figure 4B). Moreover, a lot of genes were highly expressed in whole-seed developmental stages but expressed at low levels in the shell (Figure 4B). These findings indicated that the *AhFAR1* genes might be involved in shell or seed development in peanut. Unequal expression of the *AhFAR1* genes between the two subgenomes (At > Bt) was observed (Figure 4C) indicating a divergence of gene biological function compared to in other polyploid plants (Wu et al., 2018). GO term enrichment analysis revealed that the *AhFAR1* genes are mainly involved ion, nucleic acid, and compound binding (Figure 4D).

Previous reports indicated that the *FAR1* gene family is involved in the light response in plants (Casal, 2000; Wang and Deng, 2003; Huang et al., 2012), and expanded in *A. hypogaea*, *A. duranensis*, and *A. ipaensis* (Chen et al., 2016a; Lu et al., 2018; Yan et al., 2020). From these findings, we hypothesized that the *FAR1* genes might be related to the peanut’s unique pod development pattern, such as its aerial flower and subterranean fruit. After pollination, the ovule-carrying peg (P0 stage) forms and elongates with peg-bending growth to bury the fertilized ovule into the soil (P1 stage), and then the pod develops continuously until maturation (P2–P10 stages) (Figure 4E). In this study, seven *AhFAR1* members were specifically expressed in peg and pod (shell or seed) as identified through the transcriptome analyses (Supplementary Figure 7 and Figures 4A,B). The quantitative real-time PCR (qRT-PCR) in *A. hypogaea* cv. Fuhuasheng confirmed that three of the seven *AhFAR1* genes, including *Ahy_A10g049543*, *Ahy_A06g026579*, and *Ahy_A10g048401*, had tissue-specific expression, especially in the pod and peg (Figure 4F). Moreover, examination of the five different development stages of ovule-carrying peg revealed that *Ahy_A10g049543* was highly expressed in middle and later stages but the other two genes possessed the opposite pattern (Figure 4G). In addition, the relative expression in the different development stages of the pod indicated that *Ahy_A10g049543* was highly expressed in the early stage of the pod, *Ahy_A06g026579* was mainly expressed in mature stage of the pod and *Ahy_A10g048401* was expressed in middle and late stages of the pod, such as early swelling, swelling, and mature stages (Figure 4H). The above results suggested that the *AhFAR1* genes showed specific expression responses in different tissues or development stages and might play an important role in regulating pod development in peanut. The biological function verification of the three candidate *AhFAR1* genes should not be ignored in future.

## Discussion

Higher plants regulate their growth and development through response to light changes by a system of photoreceptors. Red and far-red light are two key environmental factors influencing plant growth and development through a series of light signal transduction (Huang et al., 2012). The *FAR1* gene was identified as an important component of phyA-mediated far-red light signal pathway and was initially identified in *Arabidopsis*. As a crucial positive regulator in the phyA pathway, FAR1 can directly interact with the promoters of *FHY1* and *FHL*. In *Arabidopsis*, a total of 14 *FHY/FAR1* or *FRS* with high homology of gene structure, molecular morphology, and biological functions have been identified (Lin and Wang, 2004). Moreover, in tea plant (*Camellia sinensis*), cotton (*Gossypium hirsutum*) and pepper (*Capsicum annuum*) 25, 88, and 20 *FAR1* family members were identified, respectively (Yuan et al., 2018; Xia and Fang, 2020; Liu et al., 2021). In *A. hypogaea*, a *FAR1* gene, *AhJ11-FAR1-5*, which can enhance tolerance to drought stress, was isolated and cloned using a rapid-amplification of cDNA ends method (Yan et al., 2020). In this study, a total of 246 *FAR1* family members were identified in *A. hypogaea*, which is substantially more than those identified in the plants listed above. In addition, in two wild diploids progenitors, *A. duranensis* and *A. ipaensis*, and one wild allotetraploid species *A. monticola*, 94, 111, and 163 *FAR1* genes were identified, respectively (Supplementary Table 1). This may be due to the polyploidization events of *A. hypogaea* genome in plant evolution, which was confirmed by the subsequent *FAR1* gene family collinearity analyzes (Figure 3D).

According to phylogenetic analysis, the *FAR1* genes were divided into *Arachis* and *G. max* groups but this did not correspond to species composition and attributes among *Arachis* species (Figure 1B). This suggested that the *FAR1* genes have a greater differentiation between *Arachis* and *G. max* than among *Arachis* species. The differences between average *Ka/Ks* values of paralogous *FAR1* in *Arachis* and *G. max* also support the above statement (Figure 3B). The three classifications of 246 *AhFAR1* genes contained the same conserved motifs, suggesting that the *FAR1* genes have experienced a conservative evolutionary process and undergone relaxed diversification in peanut. The conserved protein motifs and gene structures are the most important molecular basis of gene biological function. In the present study, each group of *FAR1* family members had similar protein motifs and gene structure features (Figure 2 and Supplementary Figure 4). This finding is consistent with previous reports in *Arabidopsis* and tea plant (Lin and Wang, 2004; Liu et al., 2021). The prediction of the number of *cis*-acting elements indicated that the *AhFAR1* gene family contained rich regulatory elements, such as Box-4, G-box, GT1-motif, and TCT-motif (Supplementary Table 4), suggesting a wide range of biological functions of the *FAR1* family members in peanut.

Previous reports indicated that the *FAR1* genes exhibit different tissue-specific expression patterns in different plants. For example, in *Arabidopsis*, the *AtFAR1* genes were expressed in leaves, stems, and flowers (Lin and Wang, 2004). In cotton, most genes were highly expressed in leaves but not stems and torus (Yuan et al., 2018). In this study, about 36.2% of *AhFAR1* genes revealed tissue-specific expression patterns in flower, peg, leaf, root, and stem (Figure 4A), and 34.1% of genes exhibited development-stage-specific patterns in shell and seed (Figure 4B). The tissue- and stage-specific expression patterns revealed that the *AhFAR1* genes might play different biological functions in different tissues or developmental stages in peanut. The expression levels of the *AhFAR1* genes identified in At were higher than in Bt (Figure 4C), indicating that the *AhFAR1* genes in At might play a more important role in regulating plant growth and development in peanut. Previous findings revealed that *FAR1* acted positively in axillary bud outgrowth and could be involved in regulating branching and plant architecture by participating in strigolactones and cytokinins synthesis (Stirnberg et al., 2012). In addition, other reports indicated that the *FAR1* gene family was mainly involved in biotic and abiotic stresses, such as high or low temperature, drought, and salt exposure (Yuan et al., 2018; Liu et al., 2021). In our study, three candidate *AhFAR1* genes were specifically expressed in peg, flower, and during early pod development stages. The subsequent qRT-PCR results further confirmed the tissue-specific expression patterns (Figure 4F). Moreover, three selected *AhFAR1* genes were specifically expressed in different development stages of peg and pod (Figures 4G,H). These new findings indicate that the *AhFAR1* gene family might participate in pod development in peanut although gene biological function verification is essential future work.

## Conclusion

In summary, comprehensive and systematic analysis of the *FAR1* gene family in *Arachis*, including *A. duranensis*, *A. ipaensis*, *A. monticola*, and *A. hypogaea*, and *G. max* was performed in this study. Phylogenetic relationship and gene structure analyses suggest the conservation of *FAR1* gene family in different plant species. Phylogenetic comparison, *Ka/Ks* values and synteny analysis was carried out to uncover duplication, evolution, and domestication of the *FAR1* gene family. Most of *AhFAR1* genes were revealed to likely play multiple key roles in peanut growth and development, especially in pod development, by their differential expression patterns in distinct tissues and pod developmental stages. Overall, these analyses will promote the biological functional research of *AhFAR1* genes in pod development and shed light on the current understanding of the mechanisms of the gene family in peanut.

## Data Availability Statement

The original contributions presented in the study are included in the article/Supplementary Material, further inquiries can be directed to the corresponding author.

## Author Contributions

XC, XL, YH, and QL designed the study. QL, HaoL, HaiyL, SL, and HaifL performed the experiments. QL, RW, and QD analyzed the data. QL wrote the manuscript. HJ, RV, and MP improved the manuscript. All authors have read and approved the final manuscript.

## Conflict of Interest

The authors declare that the research was conducted in the absence of any commercial or financial relationships that could be construed as a potential conflict of interest.

## Publisher’s Note

All claims expressed in this article are solely those of the authors and do not necessarily represent those of their affiliated organizations, or those of the publisher, the editors and the reviewers. Any product that may be evaluated in this article, or claim that may be made by its manufacturer, is not guaranteed or endorsed by the publisher.

## Footnotes

1
http://www.hmmer.org/

2
http://pfam.xfam.org/

3
https://www.ncbi.nlm.nih.gov/cdd

4
http://smart.embl-heidelberg.de/

5
https://doi.org/10.5281/zenodo.31631

6
http://bioinformatics.psb.ugent.be/webtools/plantcare/html/

7
http://gsds.gao-lab.org/

8
https://CRAN.R-project.org/package=pheatmap

## Abbreviations

FAR1: far-red-impaired response 1
FHY1: far-red elongated hypocotyls 1
FHY3: far-red elongated hypocotyl 3
FRS: FAR1-related sequences
HMM: Hidden Markov model
CCD: conserved domain database
NJ: neighbor-joining
Ks: the synonymous substitution rate
Ka: the non-synonymous substitution rate
qRT-PCR: quantitative real-time PCR.
