# Supplementary material for: Genome-Wide Identification and Expression of FAR1 Gene Family Provide Insight Into Pod Development in Peanut (Arachis hypogaea)
Source: Front Plant Sci. 2022 May 3;13:893278. doi: 10.3389/fpls.2022.893278 (PMC9111957; doi:10.3389/fpls.2022.893278)
Supplement: Supplementary file 2 [file Image_1.PDF]

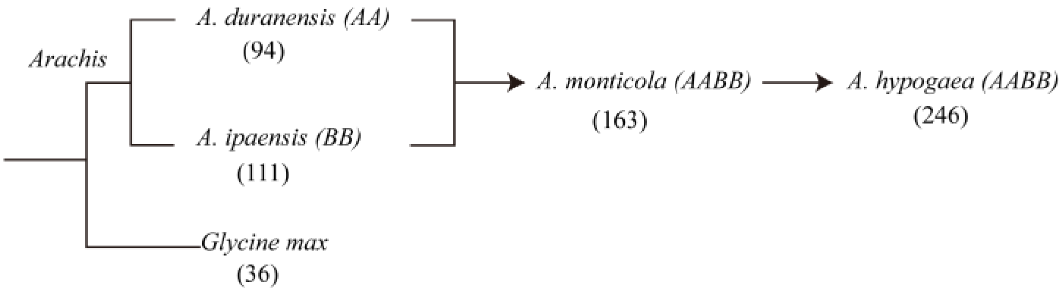

Supplementary figure 1 Evolutionary relationships of four *Arachis* species and the number of *FAR1* genes identified in each genome.

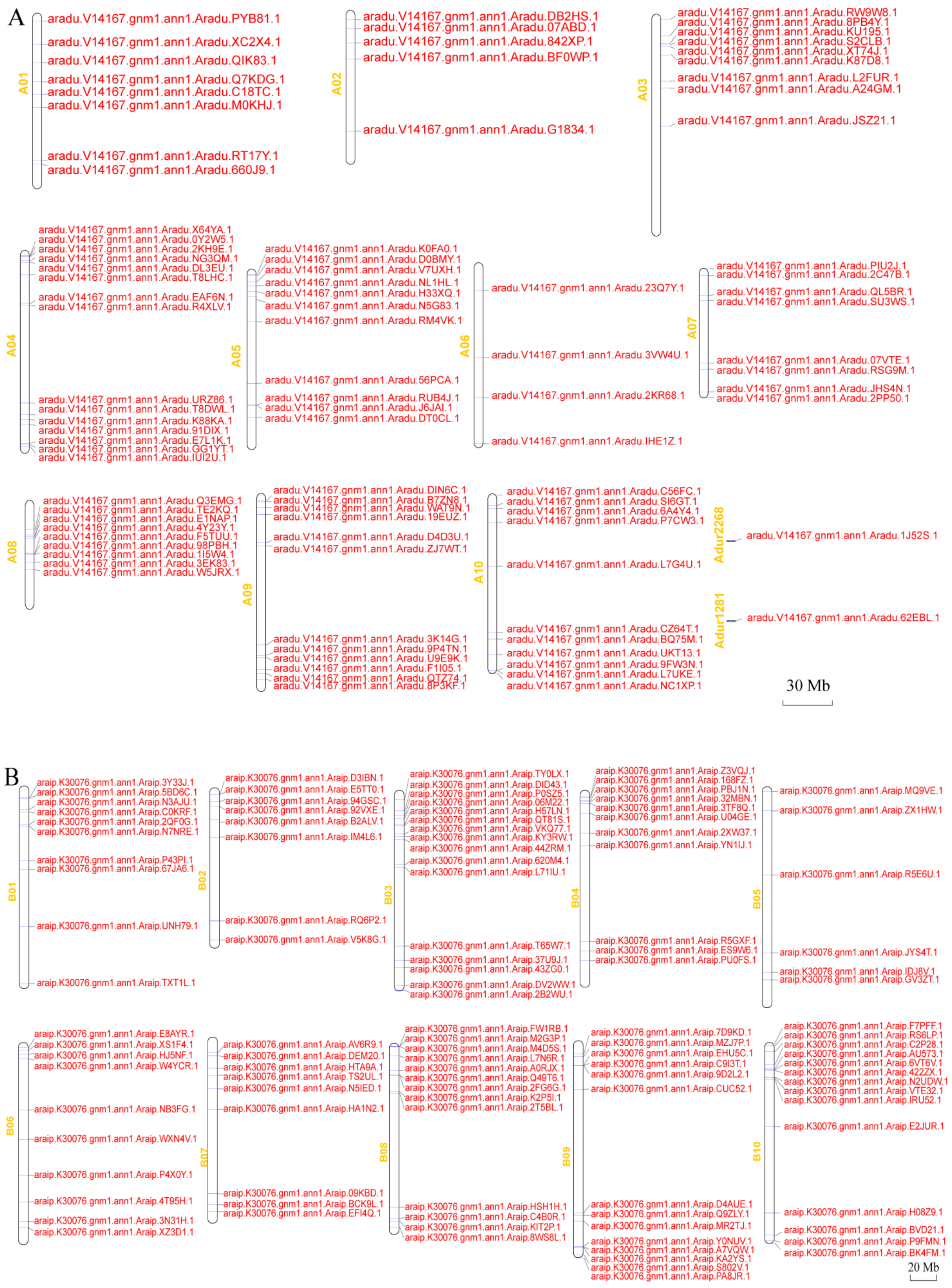
A
A01
aradu.V14167.gnm1.ann1.Aradu.PYB81.1
aradu.V14167.gnm1.ann1.Aradu.XC2X4.1
aradu.V14167.gnm1.ann1.Aradu.QIK83.1
aradu.V14167.gnm1.ann1.Aradu.Q7KDG.1
aradu.V14167.gnm1.ann1.Aradu.C18TC.1
aradu.V14167.gnm1.ann1.Aradu.M0KHJ.1
aradu.V14167.gnm1.ann1.Aradu.RT17Y.1
aradu.V14167.gnm1.ann1.Aradu.660J9.1
A02
aradu.V14167.gnm1.ann1.Aradu.DB2HS.1
aradu.V14167.gnm1.ann1.Aradu.07ABD.1
aradu.V14167.gnm1.ann1.Aradu.842XP.1
aradu.V14167.gnm1.ann1.Aradu.BF0WP.1
aradu.V14167.gnm1.ann1.Aradu.G1834.1
A03
aradu.V14167.gnm1.ann1.Aradu.RW9W8.1
aradu.V14167.gnm1.ann1.Aradu.8PB4Y.1
aradu.V14167.gnm1.ann1.Aradu.KU195.1
aradu.V14167.gnm1.ann1.Aradu.S2CLB.1
aradu.V14167.gnm1.ann1.Aradu.XT74J.1
aradu.V14167.gnm1.ann1.Aradu.K87D8.1
aradu.V14167.gnm1.ann1.Aradu.L2FUR.1
aradu.V14167.gnm1.ann1.Aradu.A24GM.1
aradu.V14167.gnm1.ann1.Aradu.JSZ21.1
A04
aradu.V14167.gnm1.ann1.Aradu.X64YA.1
aradu.V14167.gnm1.ann1.Aradu.0Y2W5.1
aradu.V14167.gnm1.ann1.Aradu.2KH9E.1
aradu.V14167.gnm1.ann1.Aradu.NG3QM.1
aradu.V14167.gnm1.ann1.Aradu.DL3EU.1
aradu.V14167.gnm1.ann1.Aradu.T8LHC.1
aradu.V14167.gnm1.ann1.Aradu.EAF6N.1
aradu.V14167.gnm1.ann1.Aradu.R4XLV.1
aradu.V14167.gnm1.ann1.Aradu.URZ86.1
aradu.V14167.gnm1.ann1.Aradu.T8DWL.1
aradu.V14167.gnm1.ann1.Aradu.K88KA.1
aradu.V14167.gnm1.ann1.Aradu.91DIX.1
aradu.V14167.gnm1.ann1.Aradu.E7L1K.1
aradu.V14167.gnm1.ann1.Aradu.GG1YT.1
aradu.V14167.gnm1.ann1.Aradu.IUI2U.1
A05
aradu.V14167.gnm1.ann1.Aradu.K0FA0.1
aradu.V14167.gnm1.ann1.Aradu.D0BMY.1
aradu.V14167.gnm1.ann1.Aradu.V7UXH.1
aradu.V14167.gnm1.ann1.Aradu.NL1HL.1
aradu.V14167.gnm1.ann1.Aradu.H33XQ.1
aradu.V14167.gnm1.ann1.Aradu.N5G83.1
aradu.V14167.gnm1.ann1.Aradu.RM4VK.1
aradu.V14167.gnm1.ann1.Aradu.56PCA.1
aradu.V14167.gnm1.ann1.Aradu.RUB4J.1
aradu.V14167.gnm1.ann1.Aradu.J6JAI.1
aradu.V14167.gnm1.ann1.Aradu.DT0CL.1
A06
aradu.V14167.gnm1.ann1.Aradu.23Q7Y.1
aradu.V14167.gnm1.ann1.Aradu.3VW4U.1
aradu.V14167.gnm1.ann1.Aradu.2KR68.1
aradu.V14167.gnm1.ann1.Aradu.IHE1Z.1
A07
aradu.V14167.gnm1.ann1.Aradu.PIU2J.1
aradu.V14167.gnm1.ann1.Aradu.2C47B.1
aradu.V14167.gnm1.ann1.Aradu.QL5BR.1
aradu.V14167.gnm1.ann1.Aradu.SU3WS.1
aradu.V14167.gnm1.ann1.Aradu.07VTE.1
aradu.V14167.gnm1.ann1.Aradu.RSG9M.1
aradu.V14167.gnm1.ann1.Aradu.JHS4N.1
aradu.V14167.gnm1.ann1.Aradu.2PP50.1
A08
aradu.V14167.gnm1.ann1.Aradu.Q3EMG.1
aradu.V14167.gnm1.ann1.Aradu.TE2KQ.1
aradu.V14167.gnm1.ann1.Aradu.E1NAP.1
aradu.V14167.gnm1.ann1.Aradu.4Y23Y.1
aradu.V14167.gnm1.ann1.Aradu.F5TUU.1
aradu.V14167.gnm1.ann1.Aradu.98PBH.1
aradu.V14167.gnm1.ann1.Aradu.1I5W4.1
aradu.V14167.gnm1.ann1.Aradu.3EK83.1
aradu.V14167.gnm1.ann1.Aradu.W5JRX.1
A09
aradu.V14167.gnm1.ann1.Aradu.DIN6C.1
aradu.V14167.gnm1.ann1.Aradu.B7ZN8.1
aradu.V14167.gnm1.ann1.Aradu.WAT9N.1
aradu.V14167.gnm1.ann1.Aradu.19EUZ.1
aradu.V14167.gnm1.ann1.Aradu.D4D3U.1
aradu.V14167.gnm1.ann1.Aradu.ZJ7WT.1
aradu.V14167.gnm1.ann1.Aradu.3K14G.1
aradu.V14167.gnm1.ann1.Aradu.9P4TN.1
aradu.V14167.gnm1.ann1.Aradu.U9E9K.1
aradu.V14167.gnm1.ann1.Aradu.F1I05.1
aradu.V14167.gnm1.ann1.Aradu.QTZ74.1
aradu.V14167.gnm1.ann1.Aradu.8P3KF.1
A10
aradu.V14167.gnm1.ann1.Aradu.C56FC.1
aradu.V14167.gnm1.ann1.Aradu.SI6GT.1
aradu.V14167.gnm1.ann1.Aradu.6A4Y4.1
aradu.V14167.gnm1.ann1.Aradu.P7CW3.1
aradu.V14167.gnm1.ann1.Aradu.L7G4U.1
aradu.V14167.gnm1.ann1.Aradu.CZ64T.1
aradu.V14167.gnm1.ann1.Aradu.BQ75M.1
aradu.V14167.gnm1.ann1.Aradu.UKT13.1
aradu.V14167.gnm1.ann1.Aradu.9FW3N.1
aradu.V14167.gnm1.ann1.Aradu.L7UKE.1
aradu.V14167.gnm1.ann1.Aradu.NC1XP.1
Adur2268
aradu.V14167.gnm1.ann1.Aradu.1J52S.1
Adur1281
aradu.V14167.gnm1.ann1.Aradu.62EBL.1
30 Mb
B
B01
araip.K30076.gnm1.ann1.Araip.3Y33J.1
araip.K30076.gnm1.ann1.Araip.5BD6C.1
araip.K30076.gnm1.ann1.Araip.N3AJU.1
araip.K30076.gnm1.ann1.Araip.C0KRF.1
araip.K30076.gnm1.ann1.Araip.2QF0G.1
araip.K30076.gnm1.ann1.Araip.N7NRE.1
araip.K30076.gnm1.ann1.Araip.P43PI.1
araip.K30076.gnm1.ann1.Araip.67JA6.1
araip.K30076.gnm1.ann1.Araip.UNH79.1
araip.K30076.gnm1.ann1.Araip.TXT1L.1
B02
araip.K30076.gnm1.ann1.Araip.D3IBN.1
araip.K30076.gnm1.ann1.Araip.E5TT0.1
araip.K30076.gnm1.ann1.Araip.94GSC.1
araip.K30076.gnm1.ann1.Araip.92VXE.1
araip.K30076.gnm1.ann1.Araip.B2ALV.1
araip.K30076.gnm1.ann1.Araip.IM4L6.1
araip.K30076.gnm1.ann1.Araip.RQ6P2.1
araip.K30076.gnm1.ann1.Araip.V5K8G.1
B03
araip.K30076.gnm1.ann1.Araip.TY0LX.1
araip.K30076.gnm1.ann1.Araip.DID43.1
araip.K30076.gnm1.ann1.Araip.P0SZ5.1
araip.K30076.gnm1.ann1.Araip.06M22.1
araip.K30076.gnm1.ann1.Araip.H57LN.1
araip.K30076.gnm1.ann1.Araip.QT81S.1
araip.K30076.gnm1.ann1.Araip.VKQ77.1
araip.K30076.gnm1.ann1.Araip.KY3RW.1
araip.K30076.gnm1.ann1.Araip.44ZRM.1
araip.K30076.gnm1.ann1.Araip.620M4.1
araip.K30076.gnm1.ann1.Araip.L71IU.1
araip.K30076.gnm1.ann1.Araip.T65W7.1
araip.K30076.gnm1.ann1.Araip.37U9J.1
araip.K30076.gnm1.ann1.Araip.43ZG0.1
araip.K30076.gnm1.ann1.Araip.DV2WW.1
araip.K30076.gnm1.ann1.Araip.2B2WU.1
B04
araip.K30076.gnm1.ann1.Araip.Z3VQJ.1
araip.K30076.gnm1.ann1.Araip.168FZ.1
araip.K30076.gnm1.ann1.Araip.PBJ1N.1
araip.K30076.gnm1.ann1.Araip.32MBN.1
araip.K30076.gnm1.ann1.Araip.3TF8Q.1
araip.K30076.gnm1.ann1.Araip.U04GE.1
araip.K30076.gnm1.ann1.Araip.2XW37.1
araip.K30076.gnm1.ann1.Araip.YN1IJ.1
araip.K30076.gnm1.ann1.Araip.R5GXF.1
araip.K30076.gnm1.ann1.Araip.ES9W6.1
araip.K30076.gnm1.ann1.Araip.PU0FS.1
B05
araip.K30076.gnm1.ann1.Araip.MQ9VE.1
araip.K30076.gnm1.ann1.Araip.ZX1HW.1
araip.K30076.gnm1.ann1.Araip.R5E6U.1
araip.K30076.gnm1.ann1.Araip.JYS4T.1
araip.K30076.gnm1.ann1.Araip.IDJ8V.1
araip.K30076.gnm1.ann1.Araip.GV3ZT.1
B06
araip.K30076.gnm1.ann1.Araip.E8AYR.1
araip.K30076.gnm1.ann1.Araip.XS1F4.1
araip.K30076.gnm1.ann1.Araip.HJ5NF.1
araip.K30076.gnm1.ann1.Araip.W4YCR.1
araip.K30076.gnm1.ann1.Araip.NB3FG.1
araip.K30076.gnm1.ann1.Araip.WXN4V.1
araip.K30076.gnm1.ann1.Araip.P4X0Y.1
araip.K30076.gnm1.ann1.Araip.4T95H.1
araip.K30076.gnm1.ann1.Araip.3N31H.1
araip.K30076.gnm1.ann1.Araip.XZ3D1.1
B07
araip.K30076.gnm1.ann1.Araip.AV6R9.1
araip.K30076.gnm1.ann1.Araip.DEM20.1
araip.K30076.gnm1.ann1.Araip.HTA9A.1
araip.K30076.gnm1.ann1.Araip.TS2UL.1
araip.K30076.gnm1.ann1.Araip.N5IED.1
araip.K30076.gnm1.ann1.Araip.HA1N2.1
araip.K30076.gnm1.ann1.Araip.09KBD.1
araip.K30076.gnm1.ann1.Araip.BCK9L.1
araip.K30076.gnm1.ann1.Araip.EFI4Q.1
B08
araip.K30076.gnm1.ann1.Araip.FW1RB.1
araip.K30076.gnm1.ann1.Araip.M2G3P.1
araip.K30076.gnm1.ann1.Araip.M4D5S.1
araip.K30076.gnm1.ann1.Araip.L7N6R.1
araip.K30076.gnm1.ann1.Araip.A0RJX.1
araip.K30076.gnm1.ann1.Araip.Q49T6.1
araip.K30076.gnm1.ann1.Araip.2FG6G.1
araip.K30076.gnm1.ann1.Araip.K2P5I.1
araip.K30076.gnm1.ann1.Araip.2T5BL.1
araip.K30076.gnm1.ann1.Araip.HSH1H.1
araip.K30076.gnm1.ann1.Araip.C4B0R.1
araip.K30076.gnm1.ann1.Araip.KIT2P.1
araip.K30076.gnm1.ann1.Araip.8WS8L.1
B09
araip.K30076.gnm1.ann1.Araip.7D9KD.1
araip.K30076.gnm1.ann1.Araip.MZJ7P.1
araip.K30076.gnm1.ann1.Araip.EHU5C.1
araip.K30076.gnm1.ann1.Araip.C9I3T.1
araip.K30076.gnm1.ann1.Araip.9D2L2.1
araip.K30076.gnm1.ann1.Araip.CUC52.1
araip.K30076.gnm1.ann1.Araip.D4AUE.1
araip.K30076.gnm1.ann1.Araip.Q9ZLY.1
araip.K30076.gnm1.ann1.Araip.MR2TJ.1
araip.K30076.gnm1.ann1.Araip.Y0NUV.1
araip.K30076.gnm1.ann1.Araip.A7VQW.1
araip.K30076.gnm1.ann1.Araip.KA2YS.1
araip.K30076.gnm1.ann1.Araip.S802V.1
araip.K30076.gnm1.ann1.Araip.PA8JR.1
B10
araip.K30076.gnm1.ann1.Araip.F7PFF.1
araip.K30076.gnm1.ann1.Araip.RS6LP.1
araip.K30076.gnm1.ann1.Araip.C2P28.1
araip.K30076.gnm1.ann1.Araip.AU573.1
araip.K30076.gnm1.ann1.Araip.6VT6V.1
araip.K30076.gnm1.ann1.Araip.422ZX.1
araip.K30076.gnm1.ann1.Araip.N2UDW.1
araip.K30076.gnm1.ann1.Araip.VTE32.1
araip.K30076.gnm1.ann1.Araip.IRU52.1
araip.K30076.gnm1.ann1.Araip.E2JUR.1
araip.K30076.gnm1.ann1.Araip.H08Z9.1
araip.K30076.gnm1.ann1.Araip.BVD21.1
araip.K30076.gnm1.ann1.Araip.P9FMN.1
araip.K30076.gnm1.ann1.Araip.BK4FM.1
20 Mb

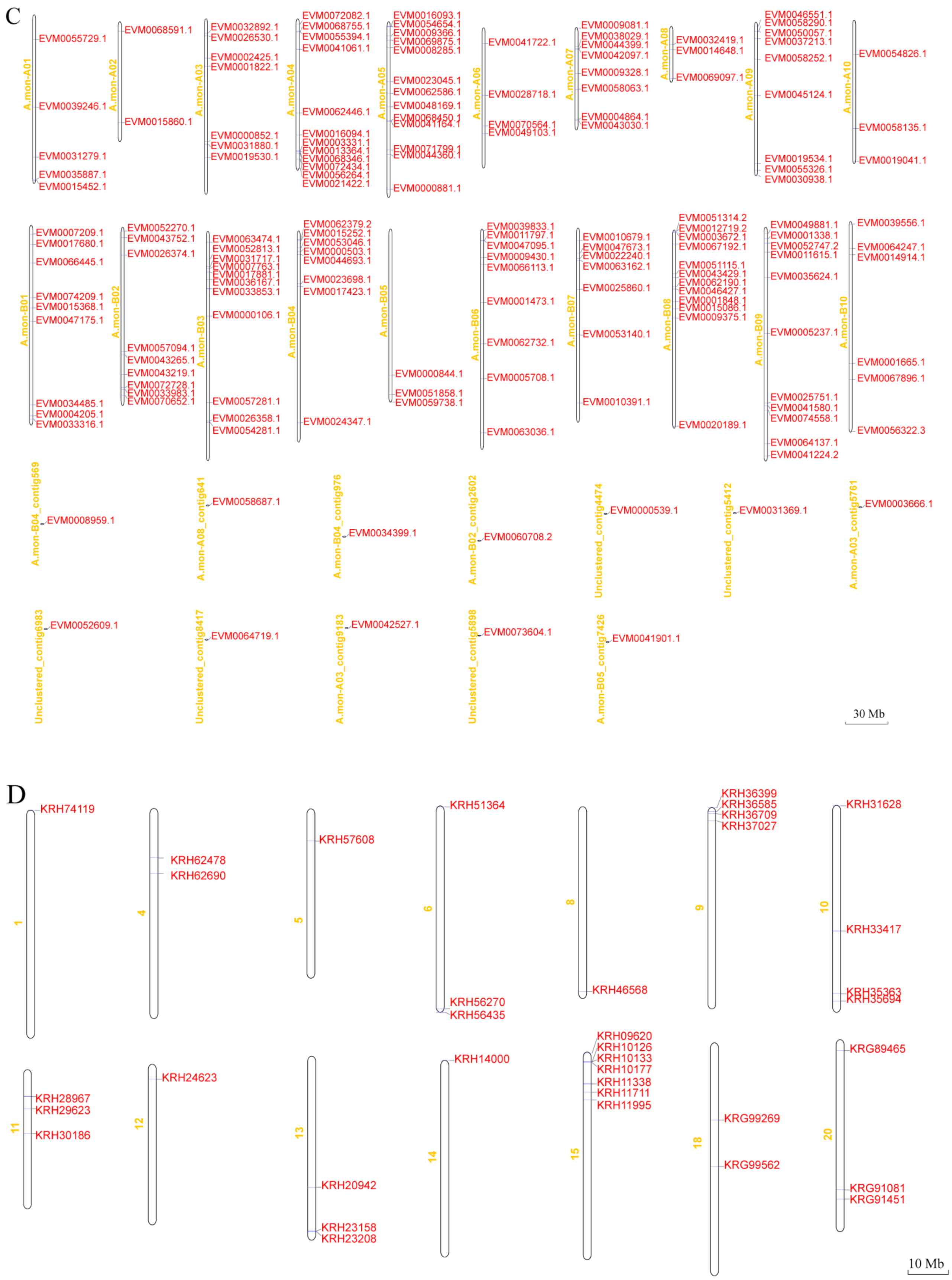

Supplementary figure 2 The genomic distribution of *FAR1* genes in *A. duranensis* (A), *A. ipaensis* (B), *A. monticola* (C) and *G. max* (D).

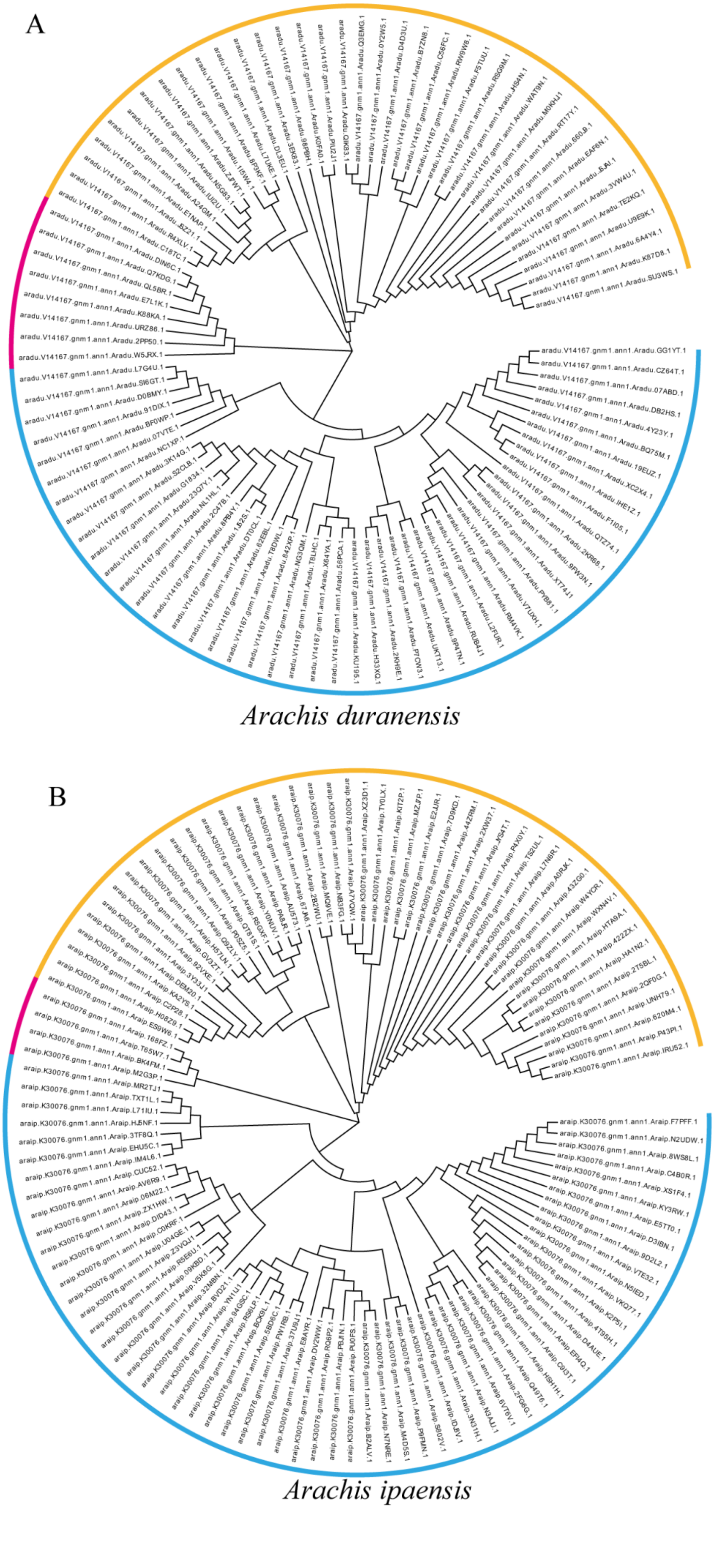
A
Arachis duranensis
B
Arachis ipaensis

C

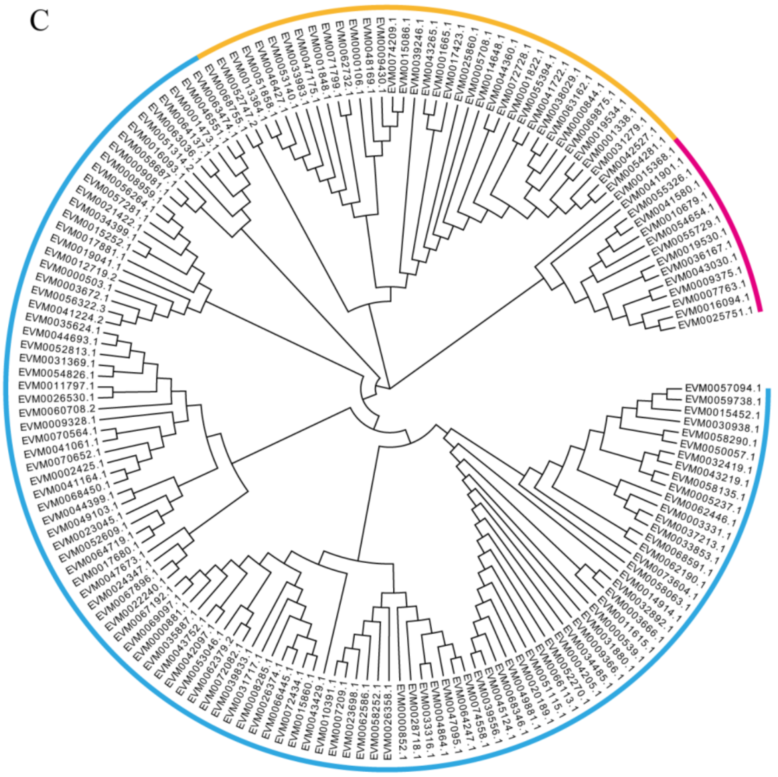

Arachis monticola

D

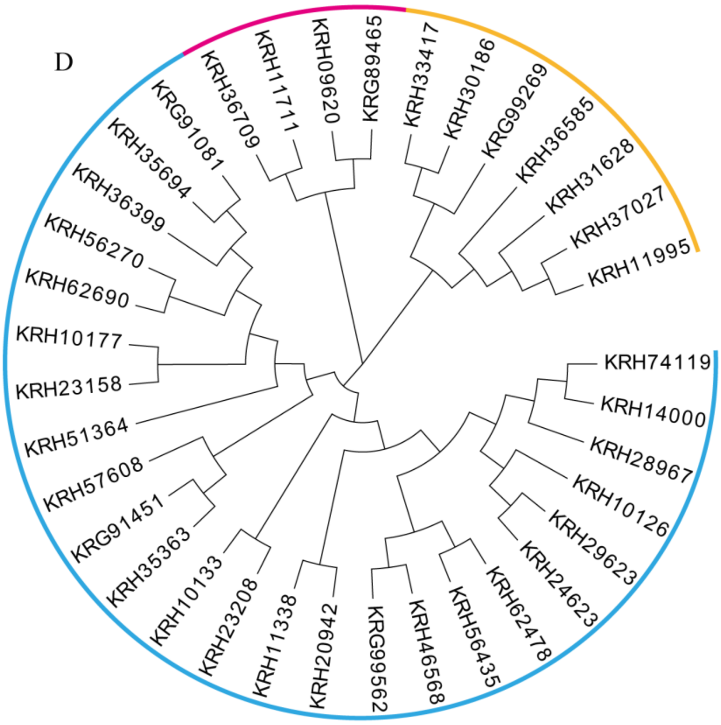

Glycine max

Supplementary figure 3 Phylogenetic tree representing relationships among FAR1 DNA binding domains of *A. duranensis* (A), *A. ipaensis* (B), *A. monticola* (C) and *G. max* (D).

A

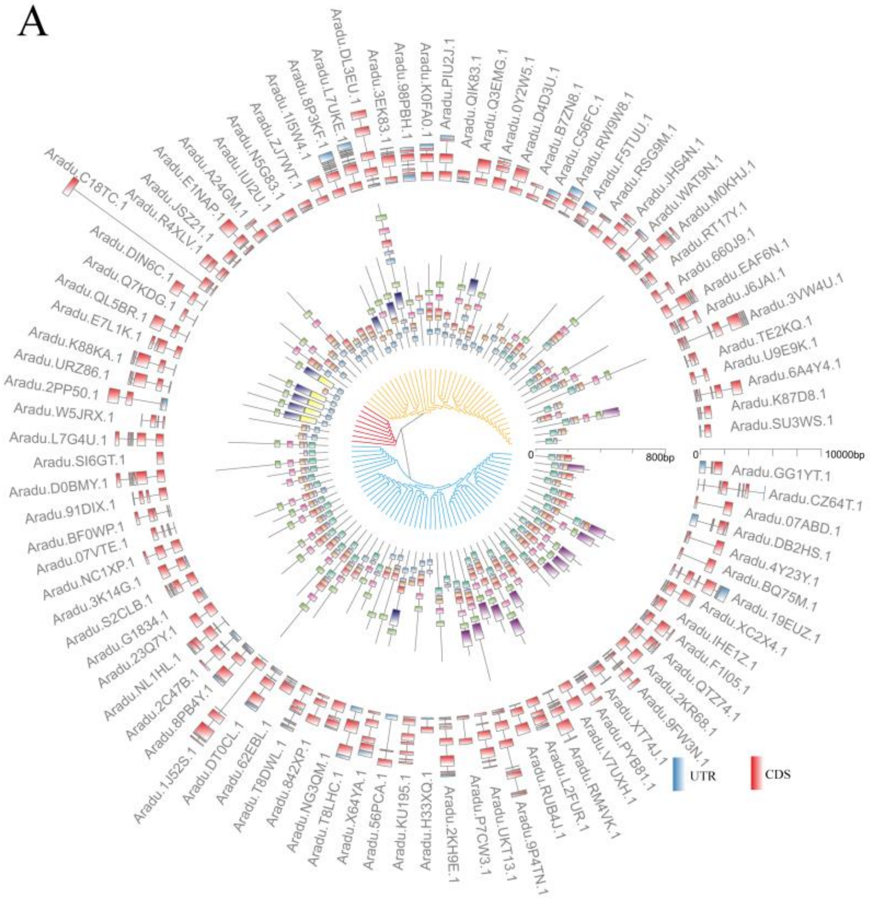

Arachis duranensis

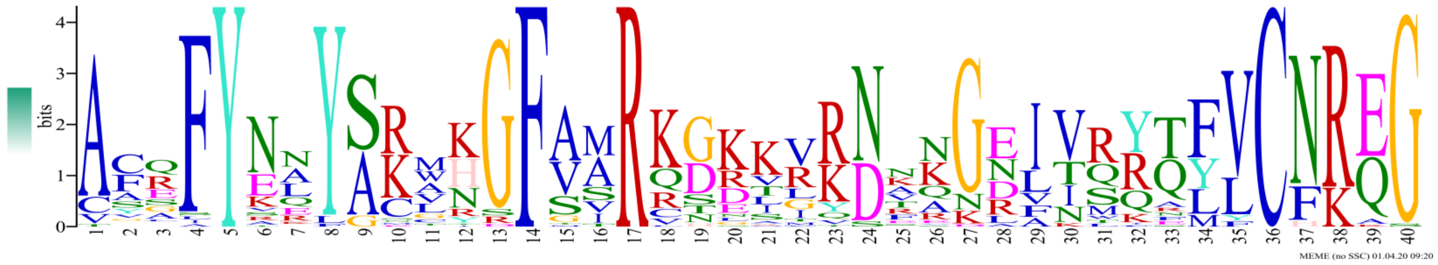

Motif 1

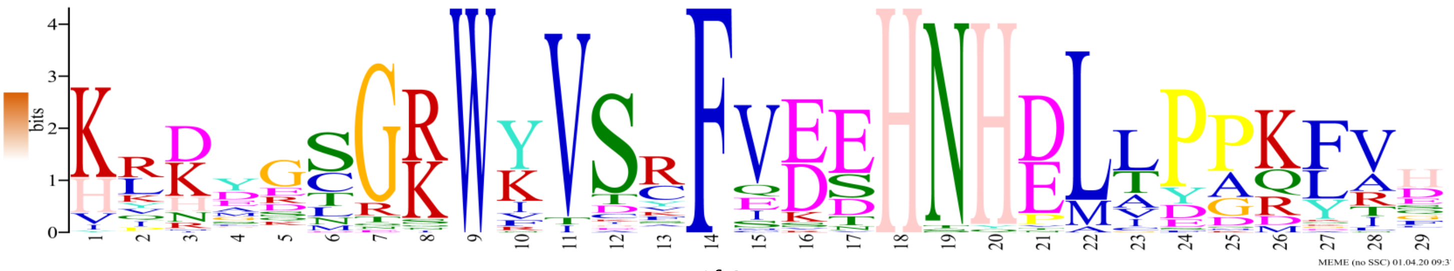

Motif 2

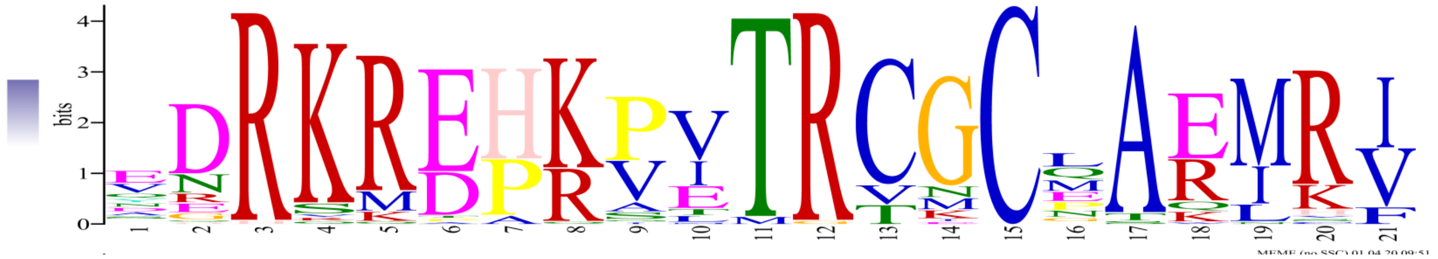

Motif 3

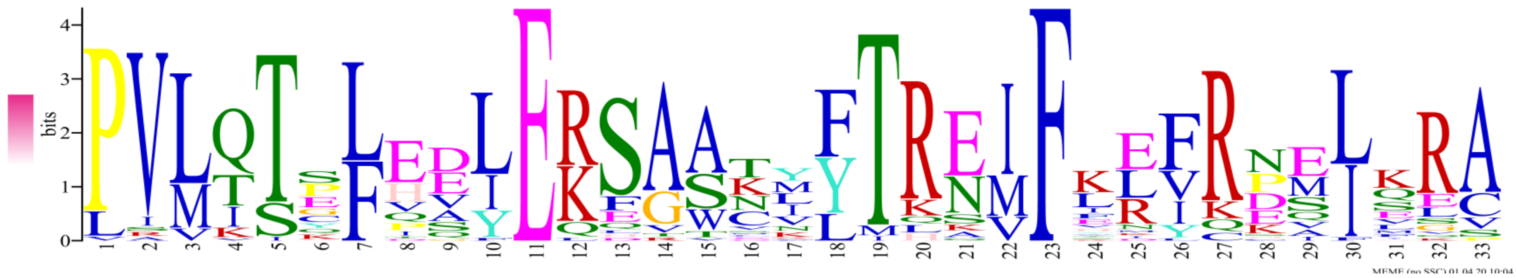

Motif 4

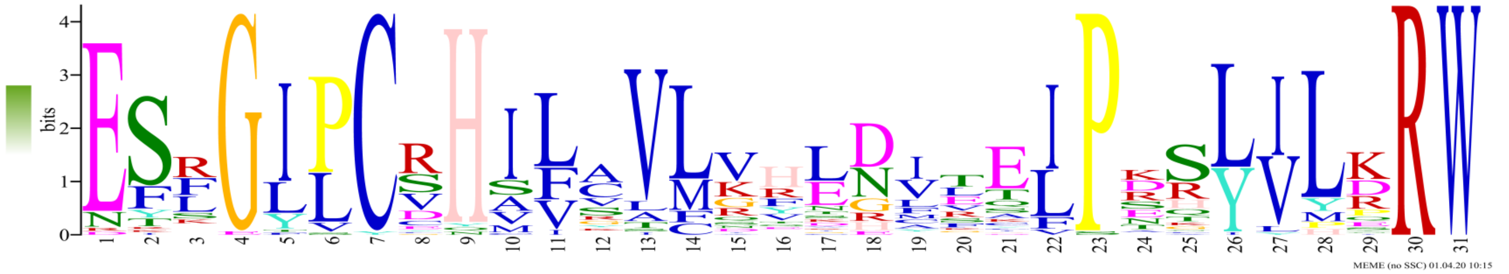

Motif 5

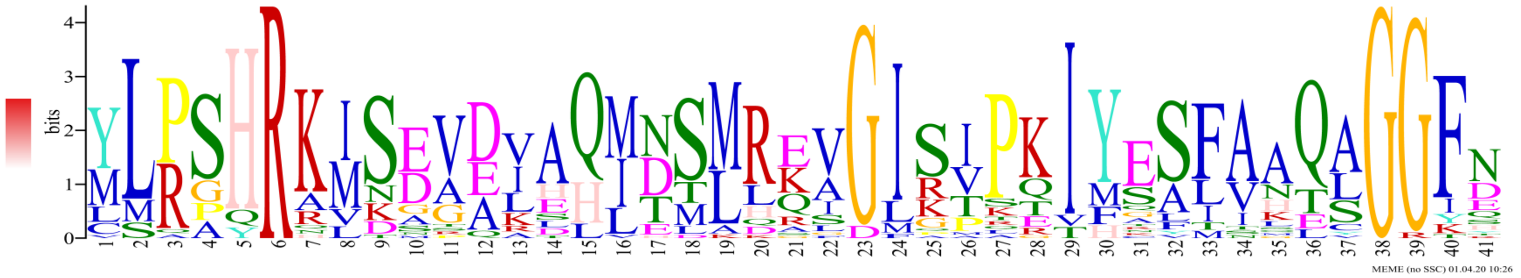

Motif 6

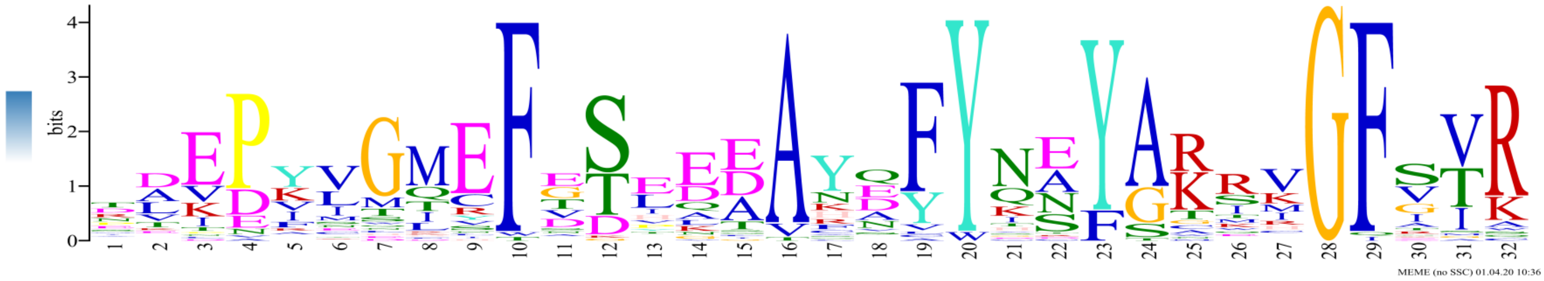

Motif 7

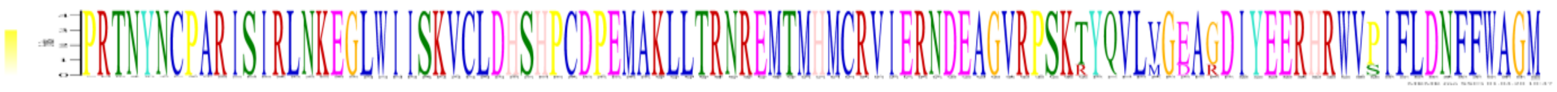

Motif 8

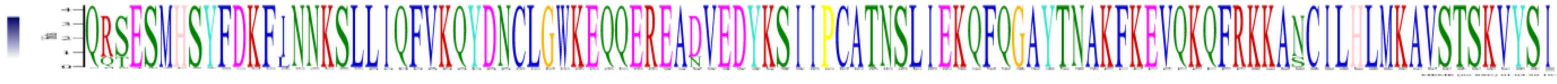

Motif 9

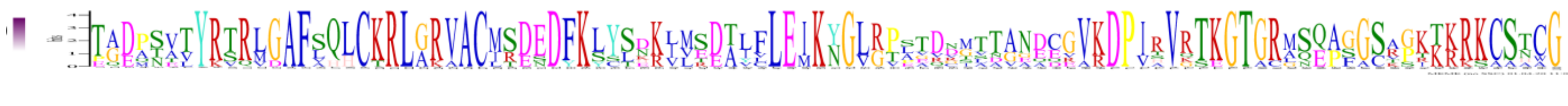

Motif 10

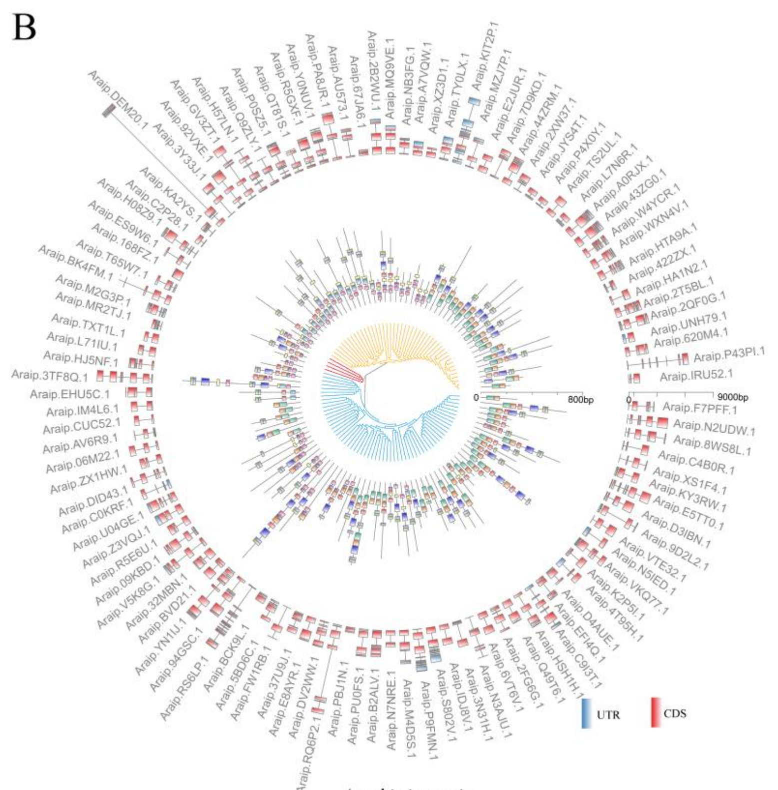

Arachis ipaensis

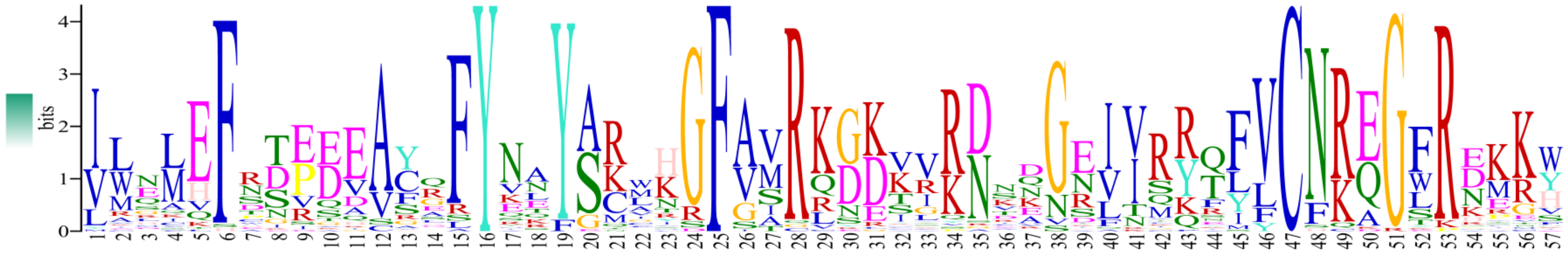

Motif 1

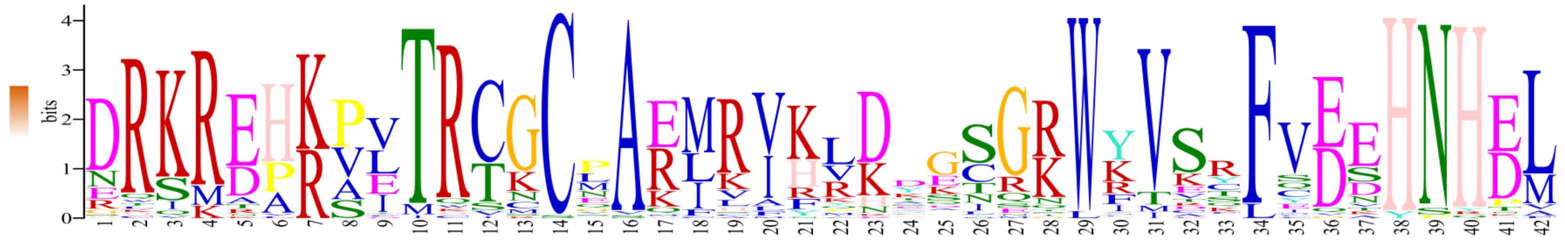

Motif 2

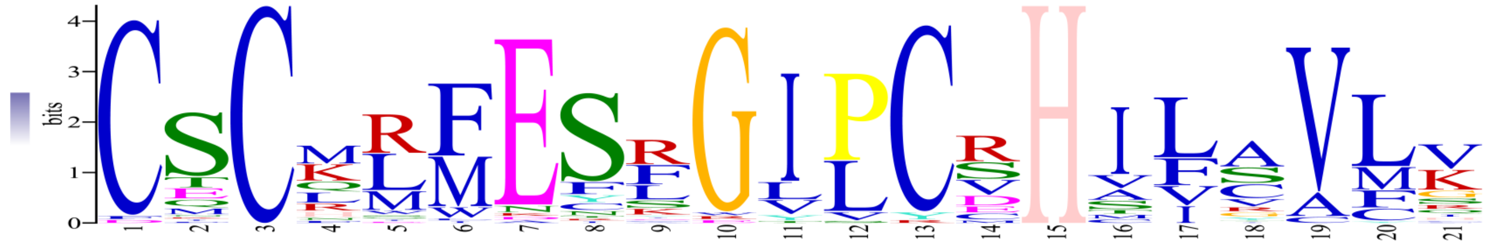

Motif 3

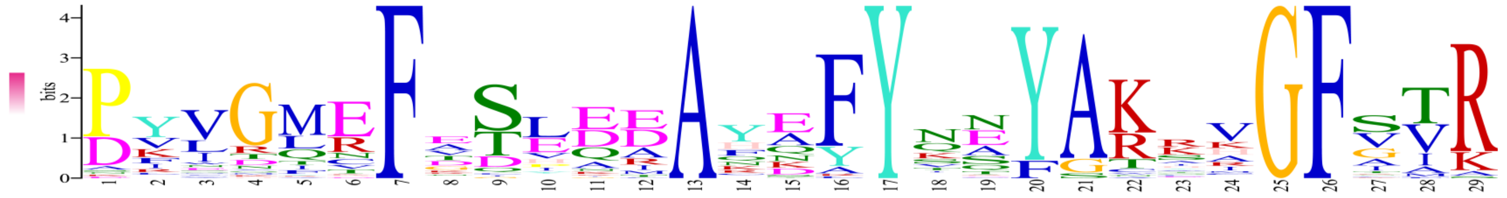

Motif 4

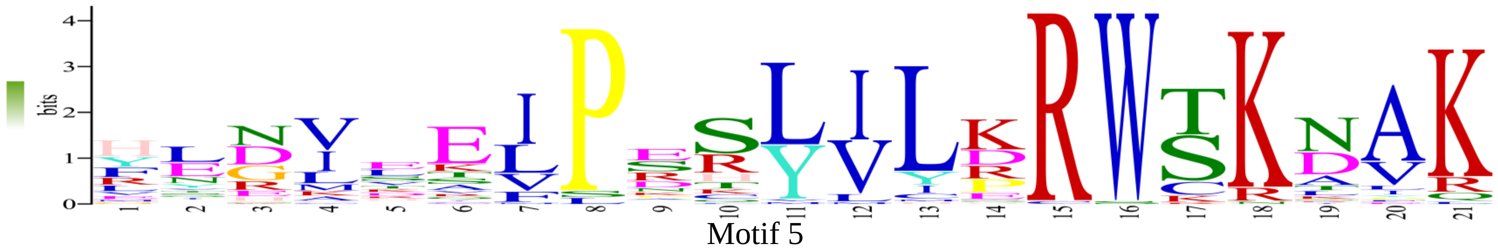

Motif 5

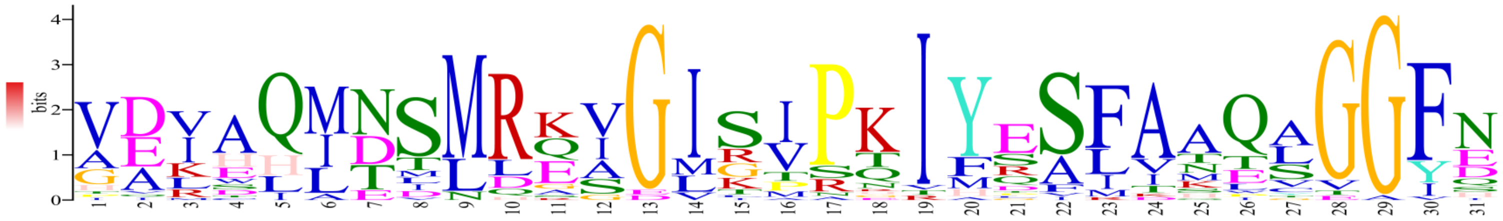

Motif 6

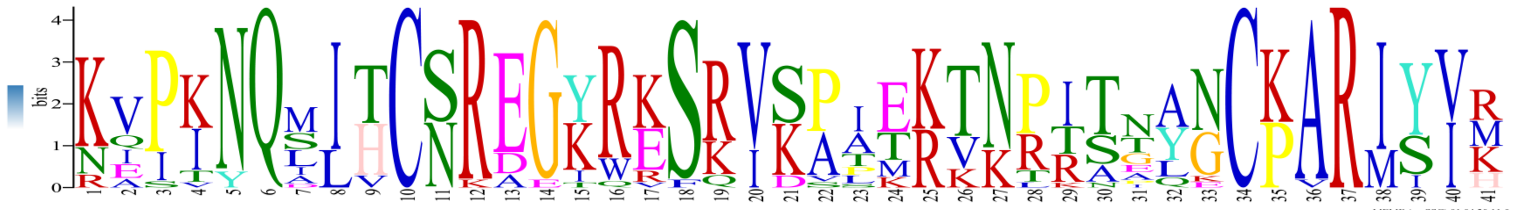

Motif 7

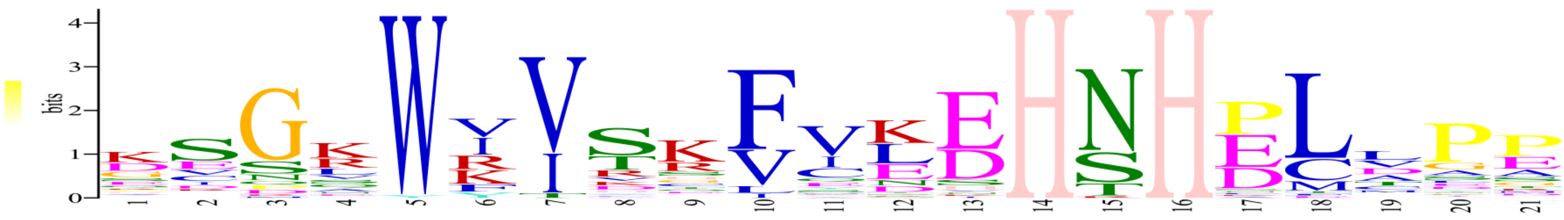

Motif 8

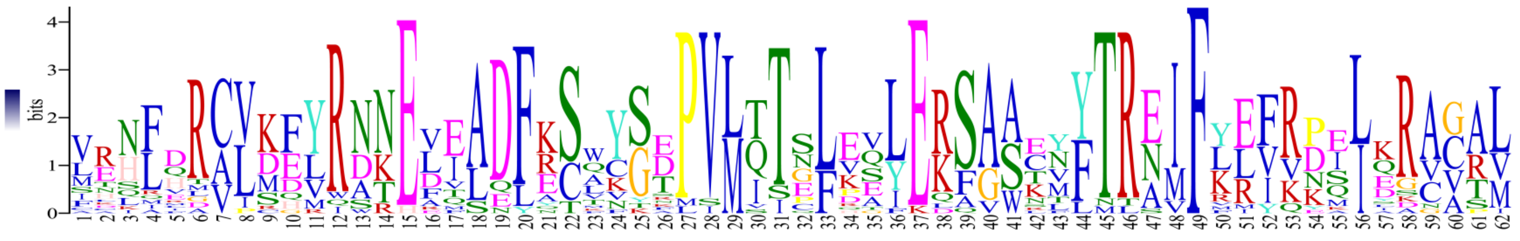

Motif 9

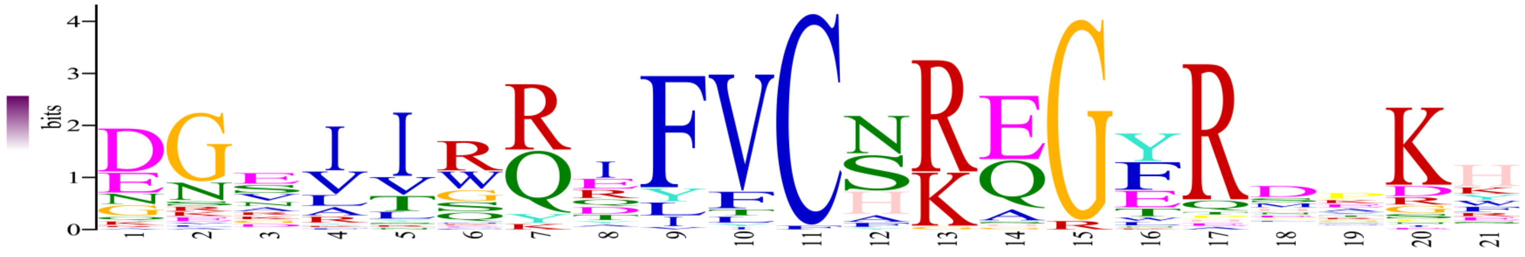

Motif 10

C

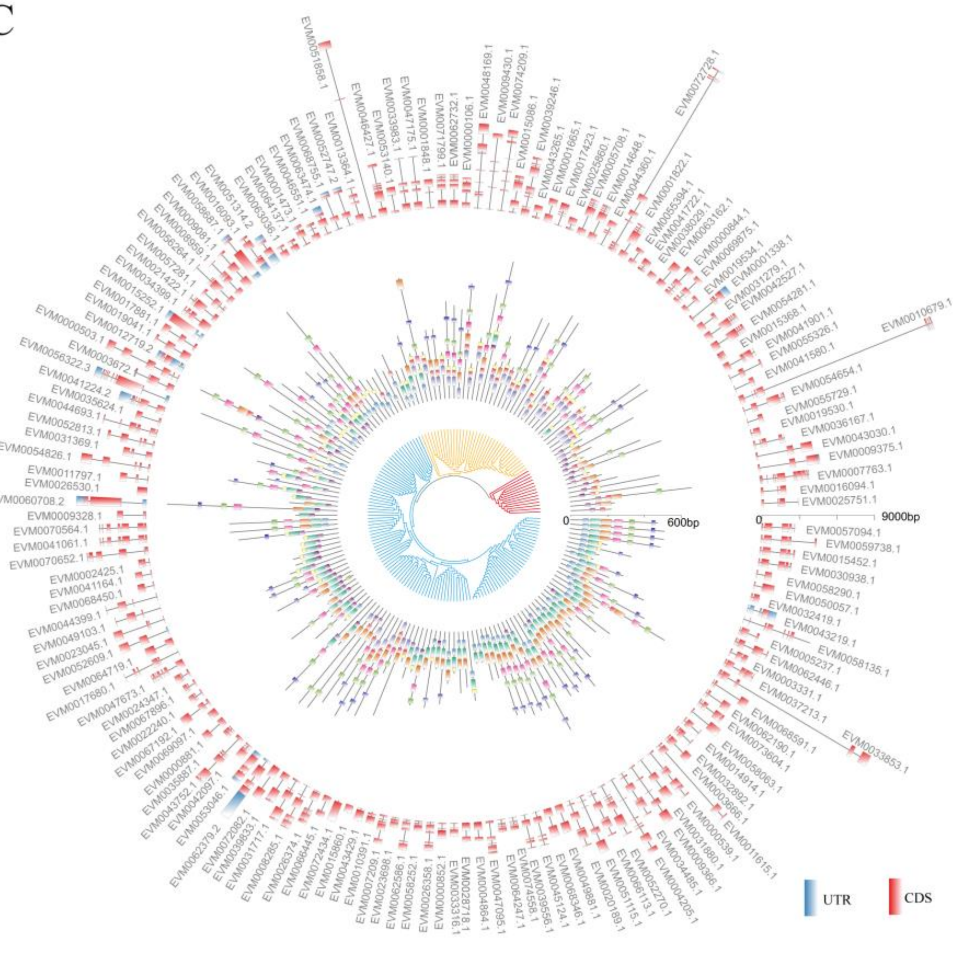

Arachis monticola

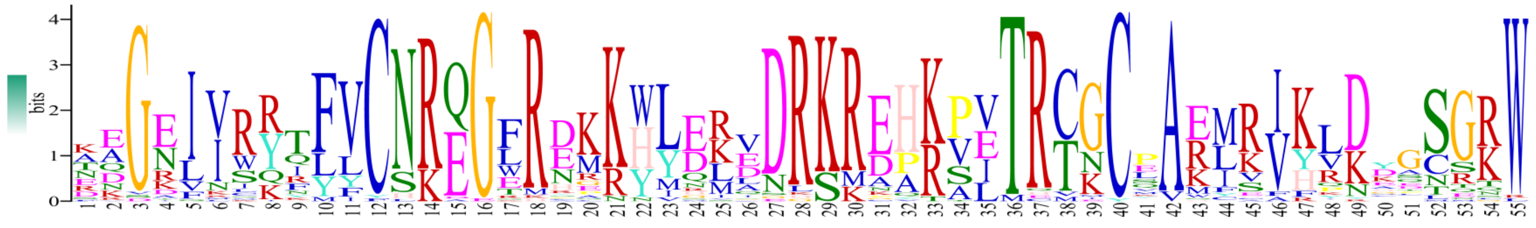

Motif 1

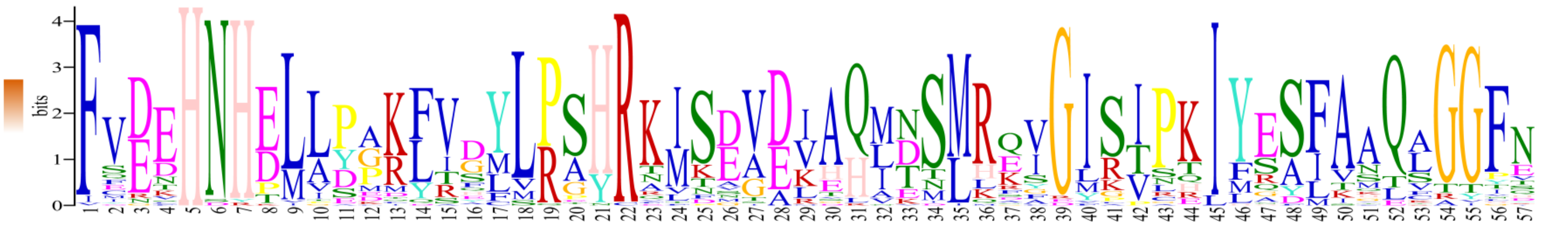

Motif 2

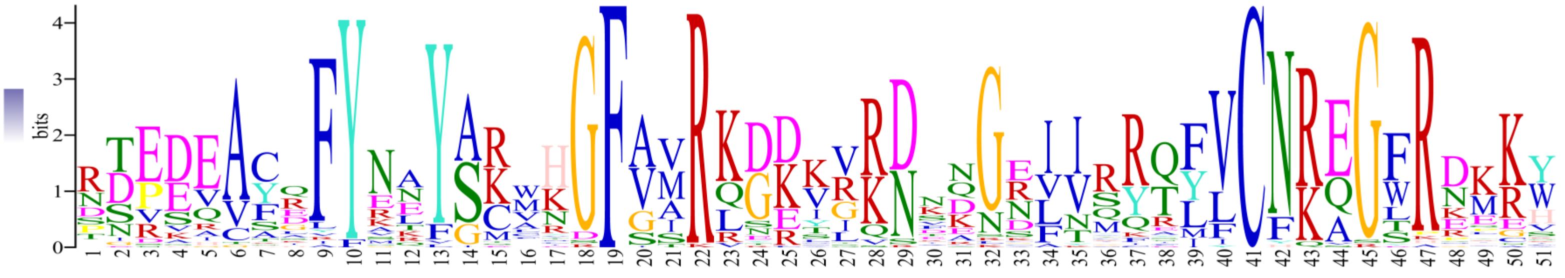

Motif 3

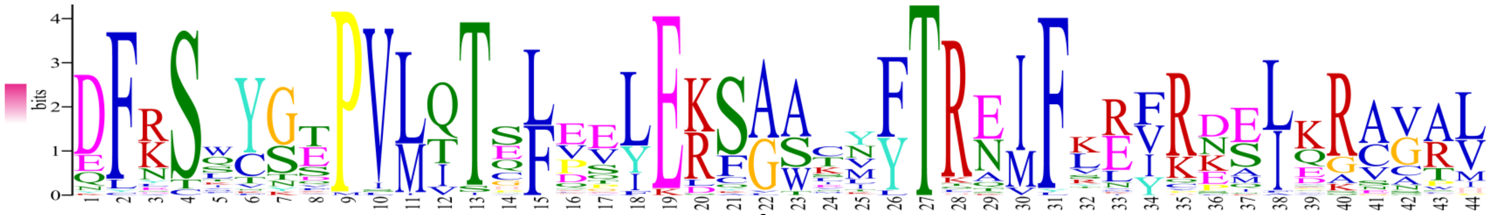

Motif 4

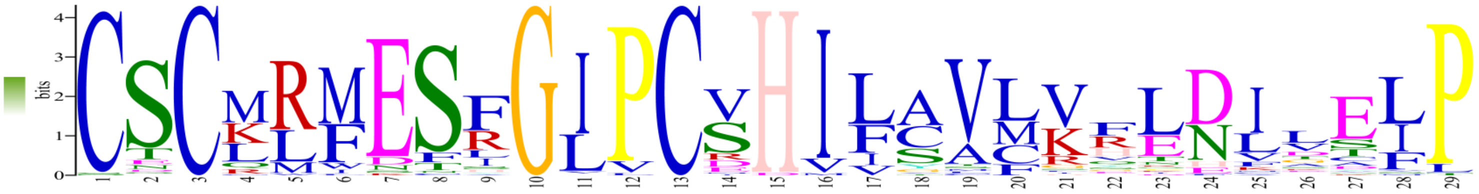

Motif 5

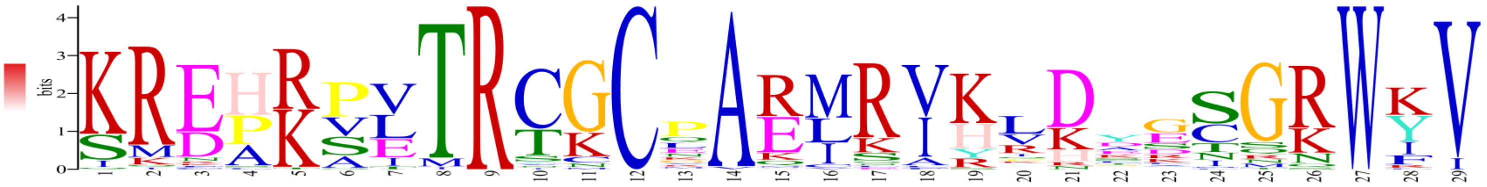

Motif 6

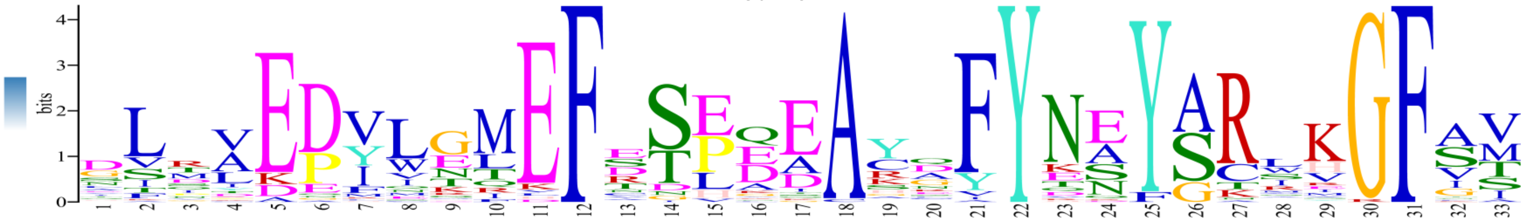

Motif 7

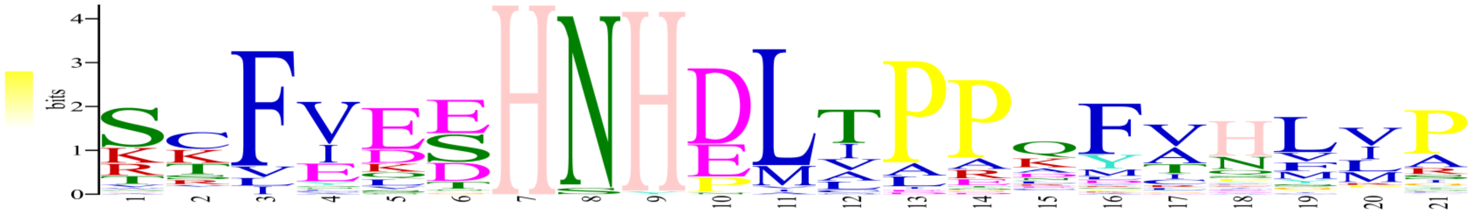

Motif 8

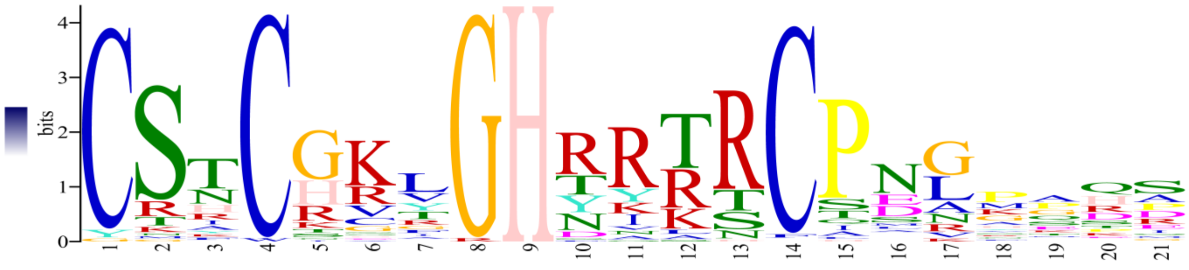

Motif 9

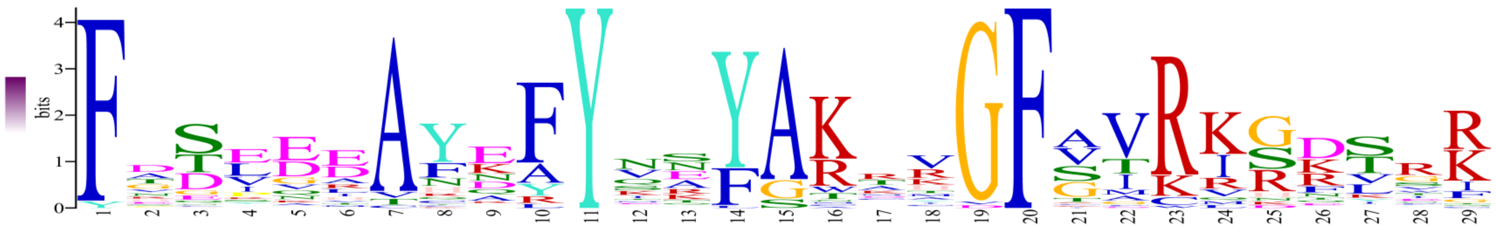

Motif 10

D

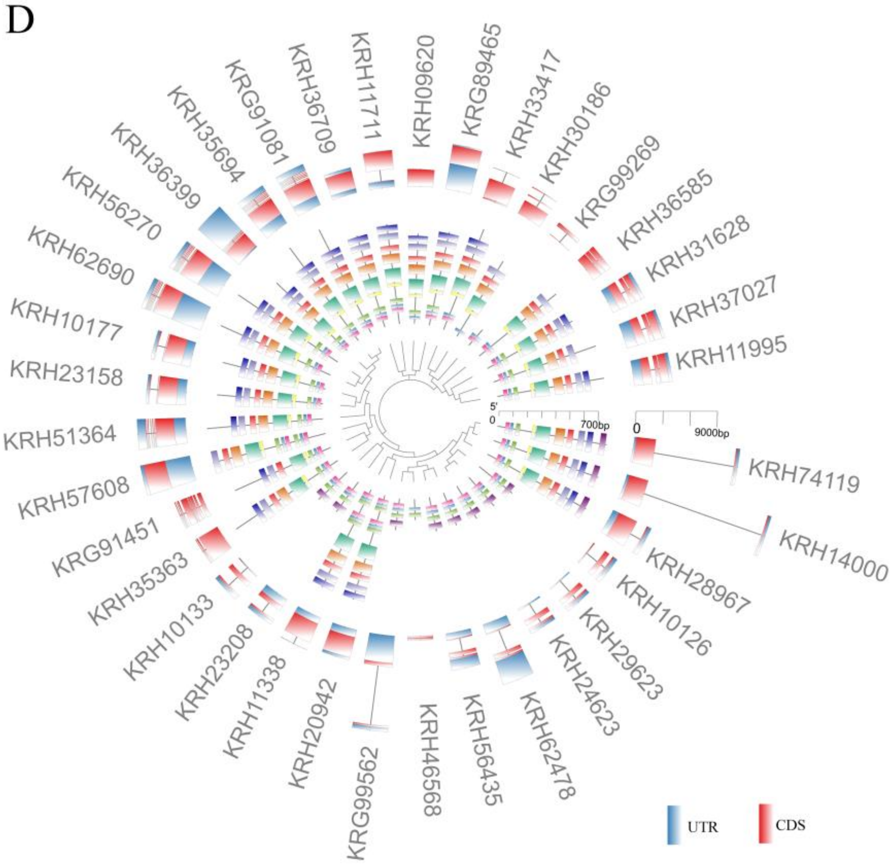

Glycine max

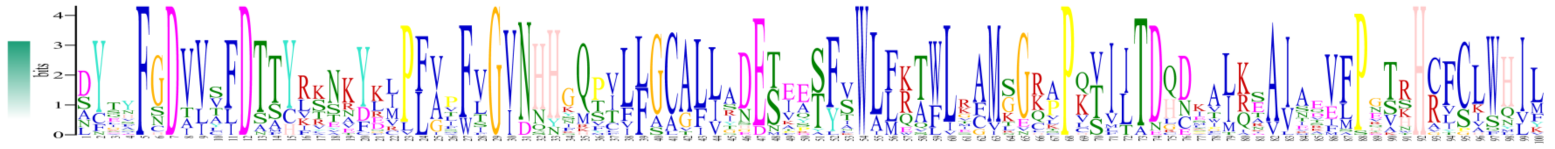

Motif 1

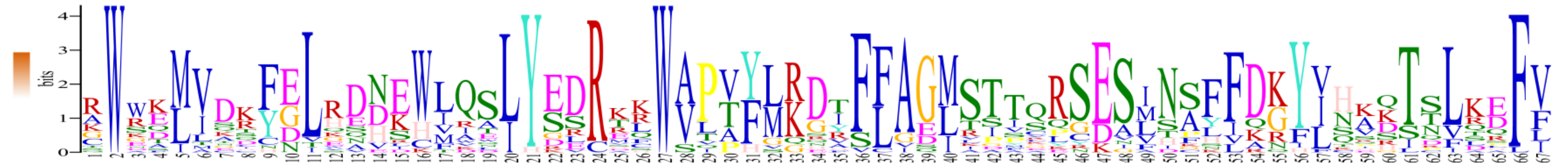

Motif 2

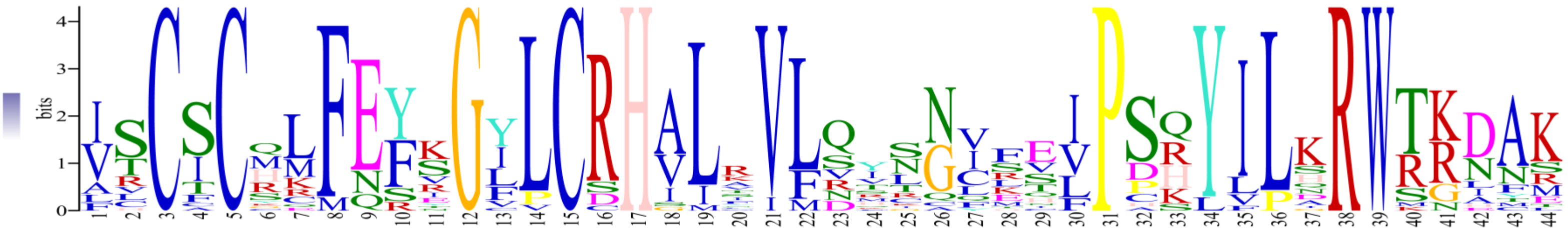

Motif 3

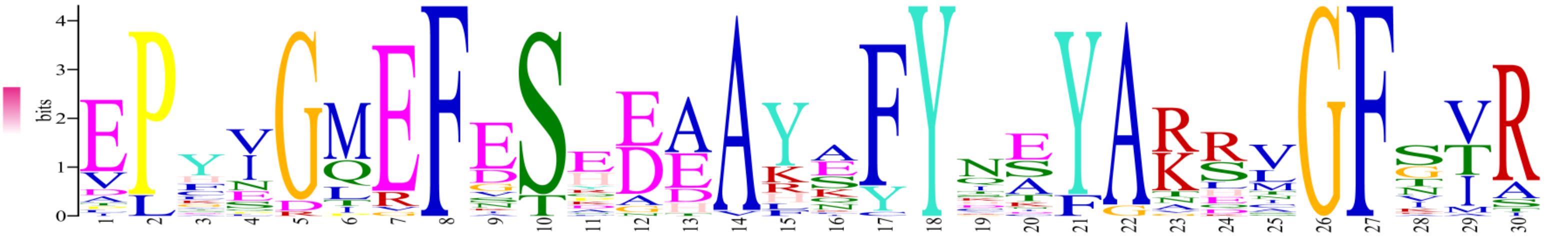

Motif 4

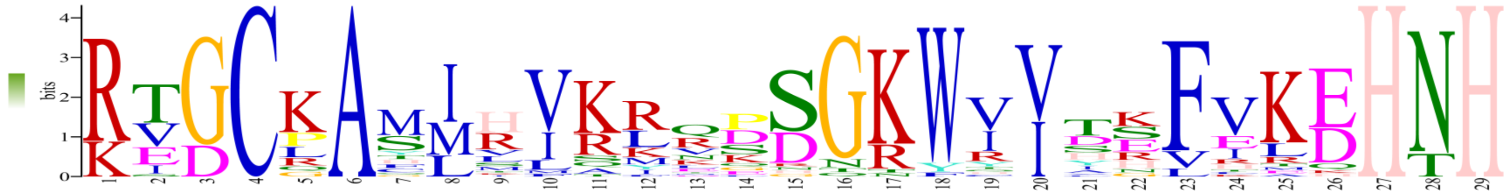

Motif 5

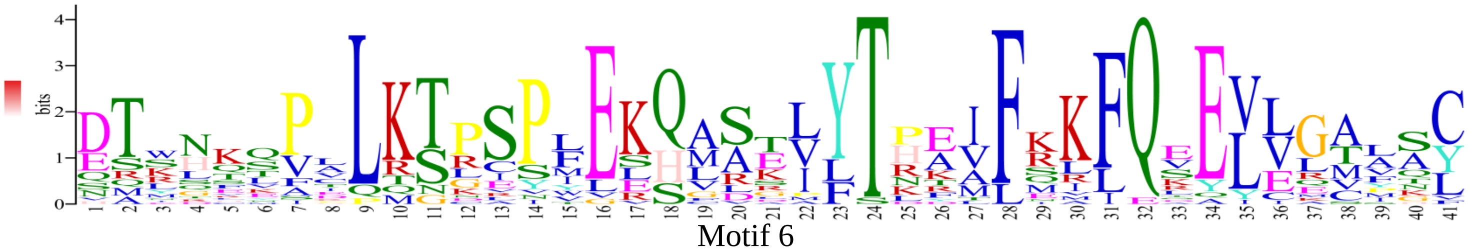

Motif 6

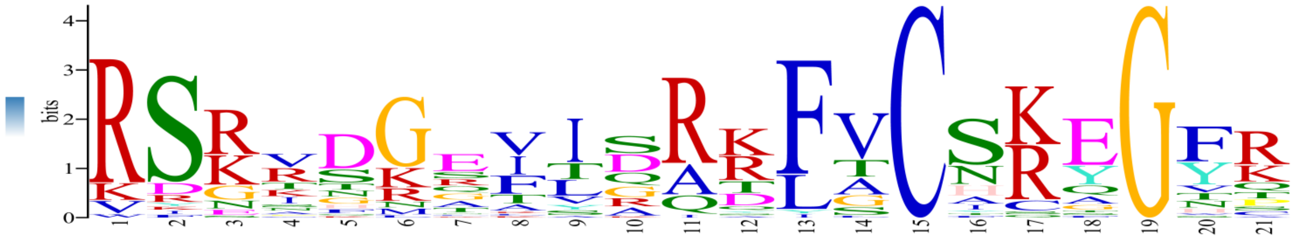

Motif 7

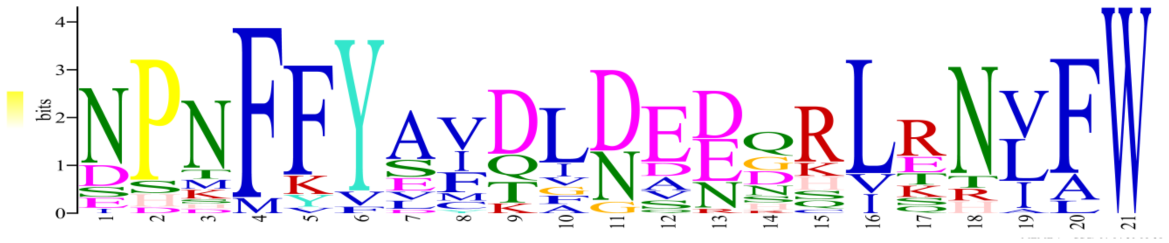

Motif 8

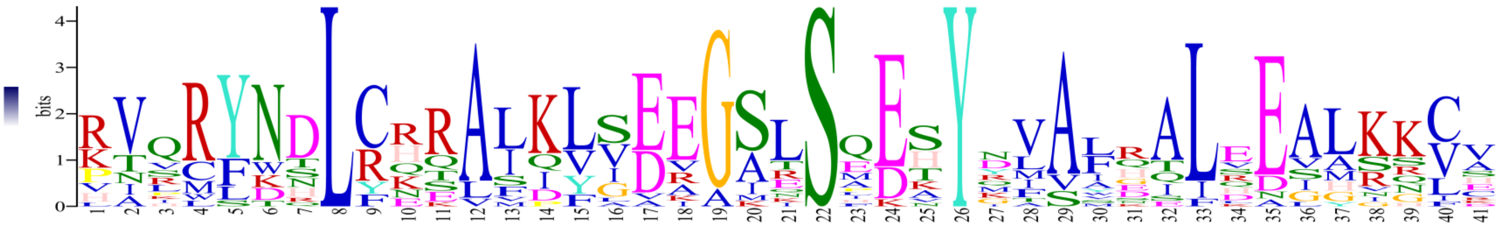

Motif 9

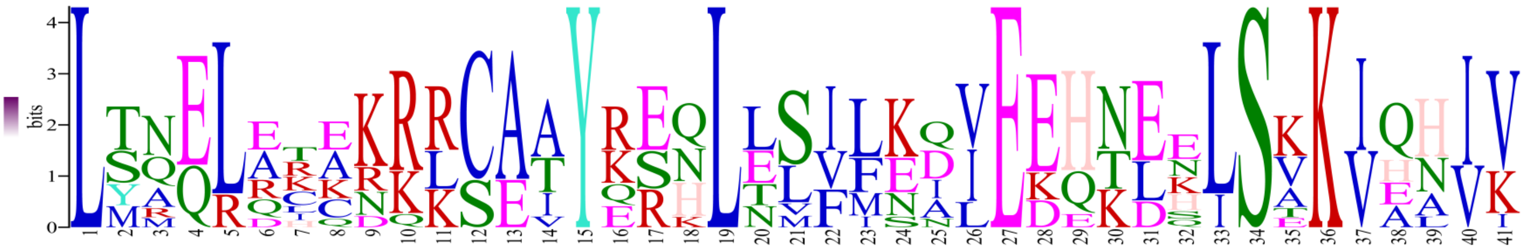

Motif 10

Supplementary figure 4 Gene structure, phylogenetic tree and motif analysis of *FAR1* gene family in *A. duranensis* (A), *A. ipaensis* (B), *A. monticola* (C) and *G. max* (D). Inner, middle and outer circle represent phylogenetic tree, motif and gene structure, respectively.

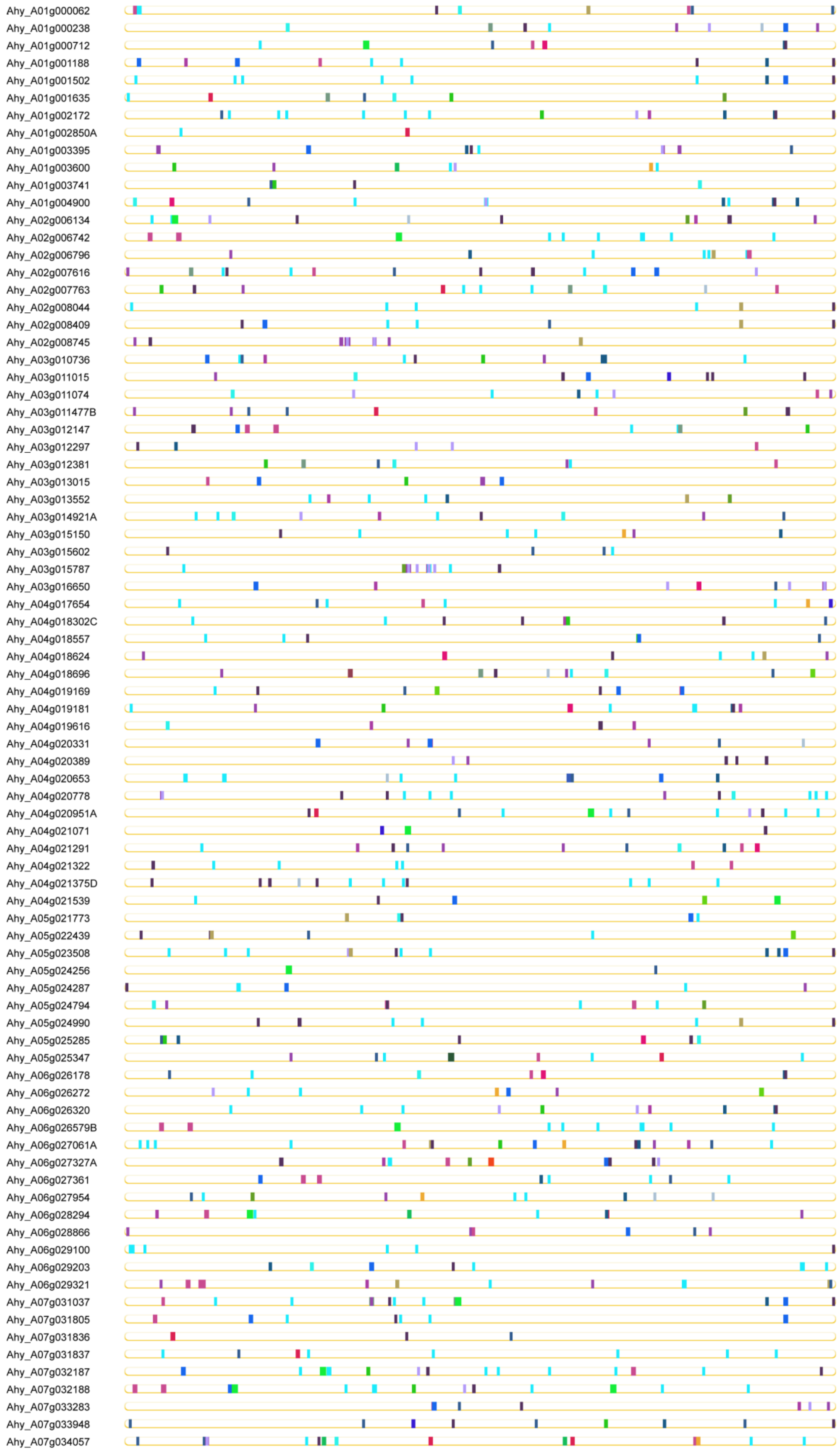

(to be continued)

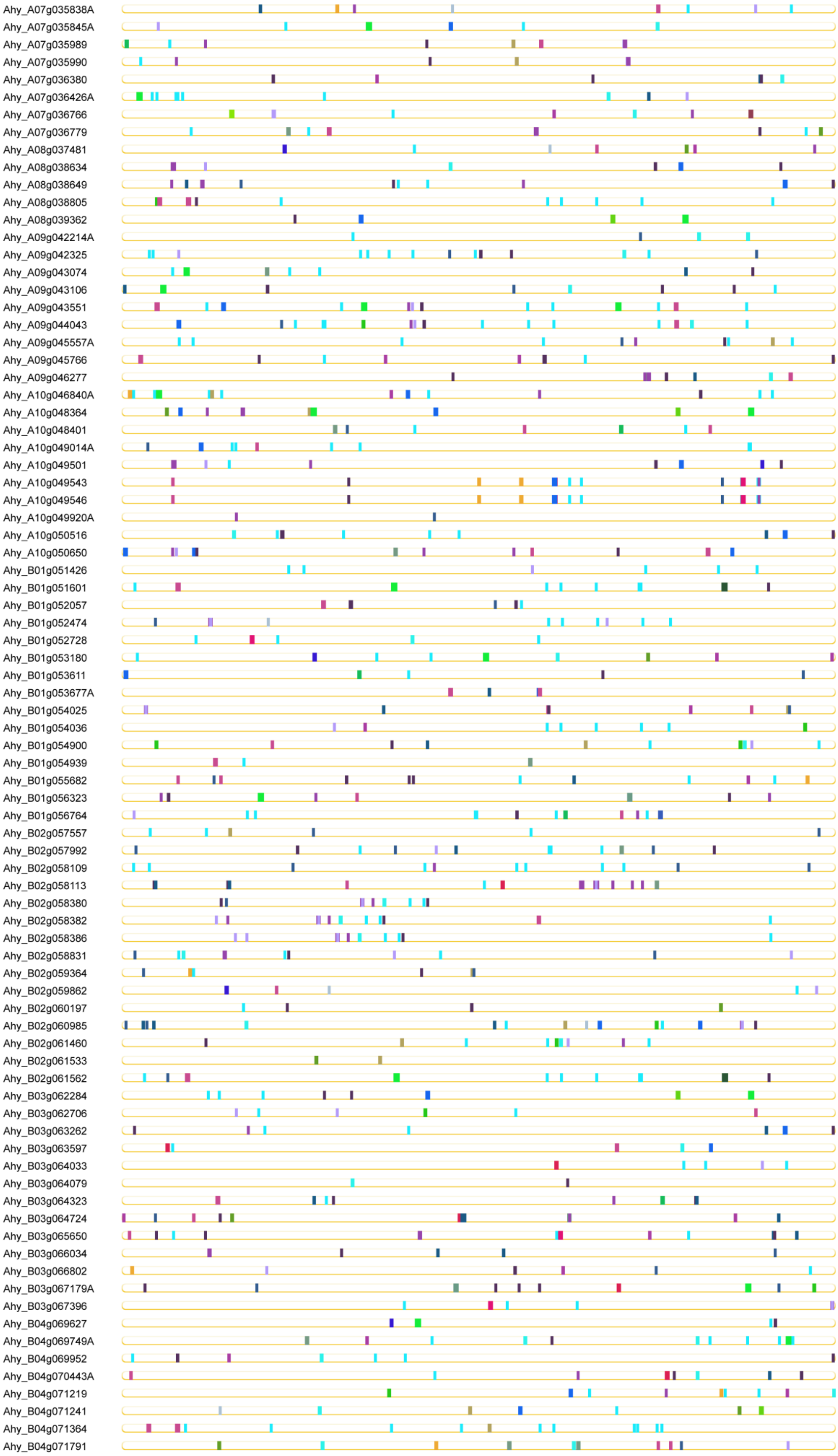

(to be continued)

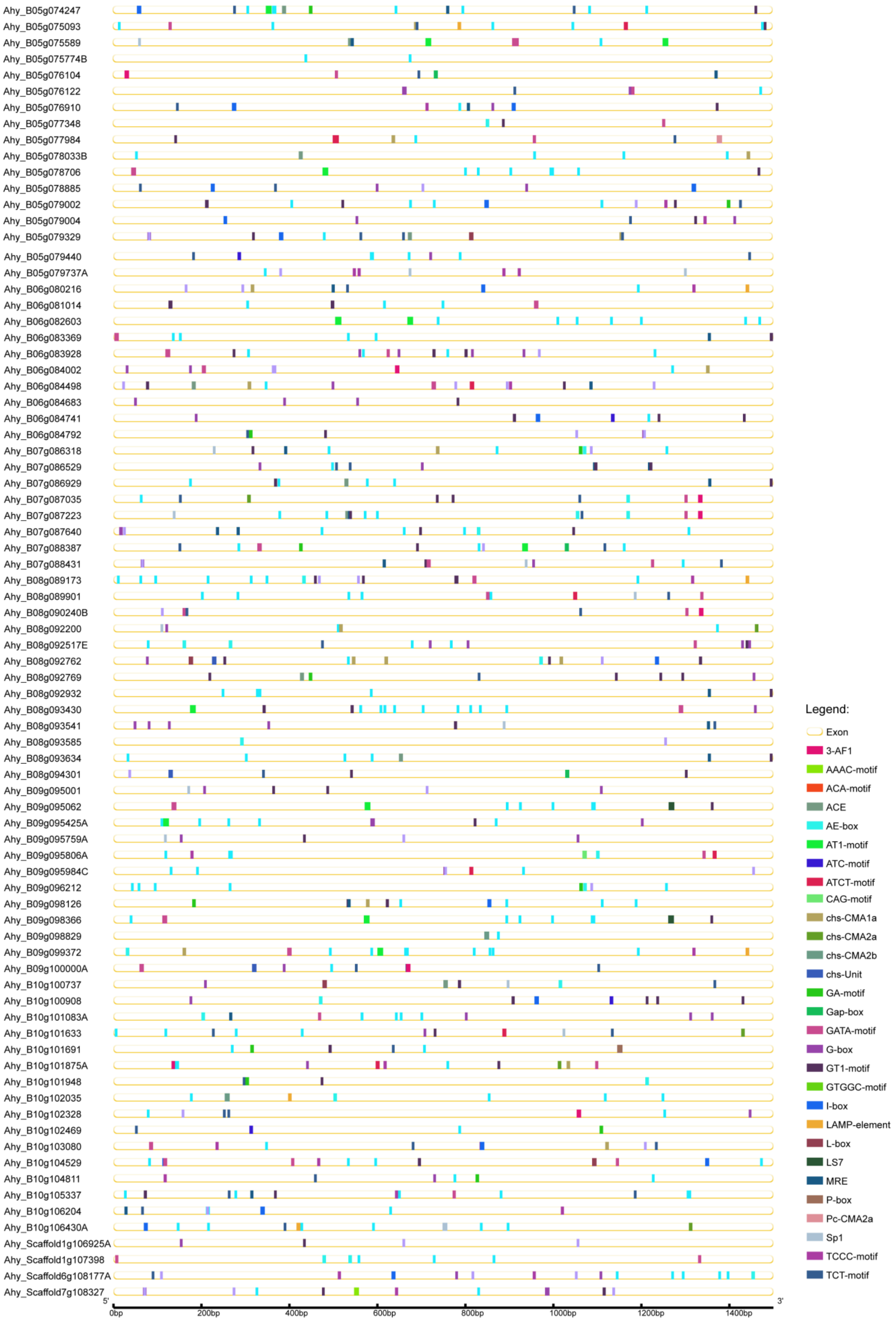

Supplementary figure 5 Distribution of *cis*-acting elements in promoter regions of *AhFAR1*.

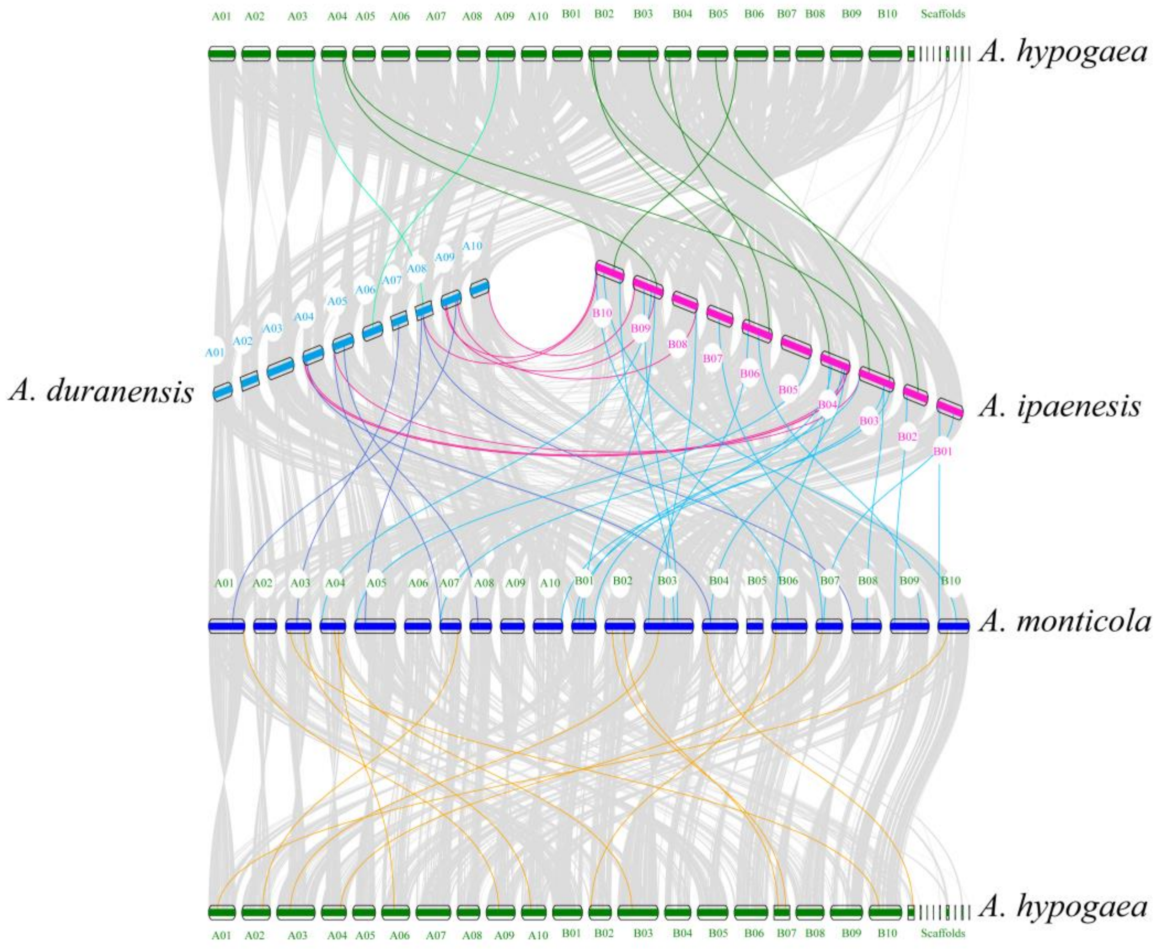

Supplementary figure 6 Synteny plot of *AhFAR1* genes among different plant speices. Gray lines represent the collinear blocks between each two plant, and red lines represent the syntenic *AhFAR1* gene pairs.

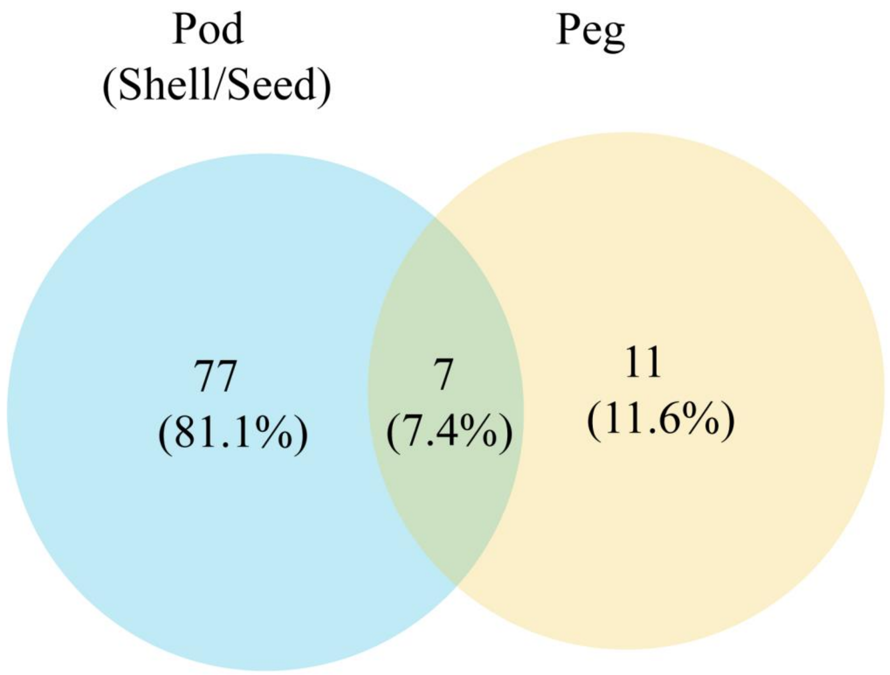

Supplementary figure 7 The number of *AhFAR1* genes specifically expressed in peg and pod.
